# Generalized bivariate Kummer-beta distribution with marginals defined on the unit interval

**DOI:** 10.1371/journal.pone.0311888

**Published:** 2024-10-28

**Authors:** Sina Shabgard, Anis Iranmanesh, Najmeh Nakhaei Rad, Daya K. Nagar, Saralees Nadarajah

**Affiliations:** 1 Department of Mathematics and Statistics, Mashhad Branch, Islamic Azad University, Mashhad, Iran; 2 Department of Statistics, University of Pretoria, Pretoria, South Africa; 3 Instituto de Matemáticas, Universidad de Antioquia, Medellín, Colombia; 4 Department of Mathematics, University of Manchester, Manchester, United Kingdom; University of Malaya, MALAYSIA

## Abstract

In this paper, a generalized bivariate Kummer-beta distribution is proposed. The name derives from the fact that its particular cases include univariate Kummer-beta distributions. This distribution generalizes a number of existing bivariate beta distributions, including Nadarajah’s bivariate distributions, Libby and Novick’s bivariate beta distribution and a central bivariate Kummer-beta distribution. Various properties associated with this newly introduced distribution are derived. The derived properties include product moments, marginal densities, marginal moments, conditional densities, conditional moments, Rényi entropy and Shannon entropy. Motivated by possible applications in economics, genetics, hydrology, meteorology, nuclear physics, and reliability, we also derive distributions of the product and the ratio of the components following the proposed distribution. Parameter estimation by maximum likelihood method is discussed by deriving expressions for score functions. Inference based on maximum likelihood estimation supposes that the maximum likelihood estimators have zero bias and zero mean squared errors. A simulation study is performed to check this for finite samples.

## 1 Introduction

The univariate beta distribution and its extensions have been generalized to the bivariate case and a number of bivariate generalizations of beta distributions are available in the scientific literature. For a review of commonly known bivariate beta distributions the reader is referred to Balakrishnan and Lai [1], Arnold, Castillo and Sarabia [2], and Kotz, Balakrishnan and Johnson [3].

The bivariate beta and associated distributions have several fields of applications. For example, bivariate beta generated distributions with classical beta marginals are natural choices as prior distributions for the parameters of correlated binomial random variables in Bayesian analysis (see Apostolakis and Moieni [4], Arnold and Ng [5]). Because of the finite range, bivariate beta distributions have many useful applications in proportions.

Perhaps, the first and the oldest bivariate generalization of the beta (type 1) distribution is the three-parameter Dirichlet distribution. Another bivariate generalization of the beta distribution is the bivariate Kummer-beta distribution studied at length by Bran-Cardona, Orozco-Castañeda and Nagar [6] and Nagar, Zarrazola and Serna-Morales [7].

While defining a three-parameter generalized beta distribution, Libby and Novick [8] also gave its multivariate (bivariate) generalization. The bivariate distributions defined and studied by Gupta, Orozco-Castañeda and Nagar [9], Jones [10], Manouchehri and Bouguila [11], Olkin and Liu [12], and Nagar, Orozco-Castañeda and Gupta [13] are particular cases of Libby and Novick’s bivariate beta distribution. Further generalizations of Libby and Novick’s bivariate beta distribution are given in Gupta and Nagar [14] and Nagar and Orozco-Castañeda [15]. Sarabia and Castillo [16] proposed several bivariate extensions of Libby and Novick’s three-parameter generalized beta type 1 distribution. Nadarajah [17, 18] reconstructed Libby and Novick’s bivariate beta distribution to define a more general bivariate distribution with generalized beta marginals and illustrated its application to drought data. In a series of papers, Nadarajah and his co-authors (Nadarajah [19, 20], Nadarajah and Kotz [21] and Nadarajah, Shih and Nagar [22]) defined four bivariate beta distributions. The normalizing constants of their pdfs involve Appell’s hypergeometric functions *F*_1_, *F*_2_, *F*_3_ and *F*_4_. Orozco-Castaẽda, Nagar and Gupta [23] derived generalized bivariate beta distributions with pdfs involving the Appell function of the second kind and the Gauss hypergeometric function. Nadarajah and Kotz [24], by using the reproductive property with respect to the distribution of the product of two independent beta variables (James [25]), proposed three bivariate beta distributions (also see Gupta and Nagar [26]). By using conditional approach (see Section 5.6 of Balakrishnan and Lai [1]), Nagar, Nadarajah and Okorie [27] constructed a bivariate distribution whose marginal laws are beta and extended beta. Recently, Olkin and Trikalinos [28] developed a bivariate beta distribution which exhibits negative as well as positive correlation.

Although many bivariate beta distributions have been defined and studied in recent years, there is always a need to develop distributions that are more flexible than existing ones and suitable for real-life data. In this paper, we define a new bivariate beta distribution with six parameters generalizing a number of existing bivariate beta distributions. We study its properties. A unique feature of the proposed distribution is that it has tails behaving polynomially and exponentially; that is, *f*(*x*, *y*)∼*Cx*^*a*^exp(−*cx*) as *x* → 0 when *y* is fixed and *f*(*x*, *y*)∼*Dy*^*b*^exp(−*dy*) as *y* → 0 when *x* is fixed. Furthermore, the parameters controlling the tail behaviours are independent for the proposed distribution which is important if data in the tails need to modeled independently. None of the known bivariate beta distributions having polynomial and exponential tails allow the parameters controlling them to be independent.

The paper is organized as follows. In Section 2, the new bivariate beta distribution is defined. We call it the *generalized bivariate Kummer-beta distribution(GBKBD)*. In Sections 3 and 4, various representations are derived for the product moments, marginal pdfs, marginal moments, conditional pdfs and conditional moments associated with the proposed bivariate distribution. In Section 5, exact forms of Rényi and Shannon entropies are derived. In Section 6, the distributions of *XY*, and $\frac{X}{Y}$ are derived when *X* and *Y* follow the bivariate beta distribution defined in this paper. In Section 7, parameter estimation by using the method of maximum likelihood is discussed. A simulation study to check the finite sample performance of the maximum likelihood estimators by MCMC methods is performed in Section 8. Finally, several results used in this paper are listed in S1 Appendix. All R codes used are given in S2 Appendix.

## 2 The generalized bivariate Kummer-beta distribution

**Definition 1** *Random variables X and Y are said to have a generalized bivariate Kummer-Beta distribution with parameters α, β, γ, σ,* λ_1_ and λ_2_, *denoted by* (*X*, *Y*) ∼ GBKB(*α*, *β*, *γ*, *σ*, λ_1_, λ_2_), *if their joint pdf is*

$$\begin{array}{c} f(x,y;\alpha ,\beta ,\gamma ,\sigma ,\lambda_{1},\lambda_{2})\\ =C(\alpha ,\beta ,\gamma ,\sigma ,\lambda_{1},\lambda_{2})\frac{x^{\alpha -1}y^{\beta -1}{\left(1-x\right)}^{\gamma -\alpha -1}{\left(1-y\right)}^{\gamma -\beta -1}}{{\left(1-\sigma xy\right)}^{\gamma }}\;\text{exp}[-(\lambda_{1}x+\lambda_{2}y)], \end{array}$$ (1)

*where 0 < x* < 1, 0 < *y* < 1, *γ* > *α* > 0, *γ* > *β* > 0, 0 ≤ *σ* < 1, −∞ < λ_1_, λ_2_ < ∞ and *C*(*α*, *β*, *γ*, *σ*, λ_1_, λ_2_) *is the normalizing constant.*

By integrating the joint pdf of *X* and *Y* over its support set, the normalizing constant can be derived as

$$\begin{array}{c} {\left[C(\alpha ,\beta ,\gamma ,\sigma ,\lambda_{1},\lambda_{2})\right]}^{-1}\\ =\int_{0}^{1}\int_{0}^{1}\frac{x^{\alpha -1}y^{\beta -1}{\left(1-x\right)}^{\gamma -\alpha -1}{\left(1-y\right)}^{\gamma -\beta -1}}{{\left(1-\sigma xy\right)}^{\gamma }}\;\text{exp}[-(\lambda_{1}x+\lambda_{2}y)]\;\text{d}x\;\text{d}y\\ =B\left(\alpha ,\gamma -\alpha \right)B\left(\beta ,\gamma -\beta \right)F_{Z}(\alpha ,\beta ,\gamma ;\gamma ,\gamma ;\sigma ,-\lambda_{1},-\lambda_{2}), \end{array}$$ (2)

where *F*_*Z*_ is a hypergeometric function of three variables defined in equation (9) in S1 Appendix.

Several special cases of (1) are worth mentioning. For *σ* = 0, the components *X* and *Y* are independent each having a Kummer-beta distribution, *X* ∼ *KB*(*α*, *γ* − *α*, λ_1_) and *Y* ∼ *KB*(*α*, *γ* − *β*, λ_2_). The pdf of *V* ∼ *KB*(*a*, *b*, *ξ*) (Nagar and Gupta [29]) is

$$\begin{array}{c} f_{\text{KB}}\left(v;a,b,\xi \right)=K\left(a,b,\xi \right)v^{a-1}{\left(1-v\right)}^{b-1}\;\text{exp}\;\left(-\xi v\right),\;0<v<1,\;a>0,\;b>0, \end{array}$$ (3)

where [*K*(*a*, *b*, *ξ*)]^−1^ = *B*(*a*, *b*) _1_*F*_1_(*a*; *a* + *b*; − *ξ*).

For λ_1_ = λ_2_ = 0, (1) reduces to a bivariate beta pdf (Nadarajah [17, 18]) given by

$$\begin{array}{c} C\left(\alpha ,\beta ,\gamma ,\sigma \right)\frac{x^{\alpha -1}y^{\beta -1}{\left(1-x\right)}^{\gamma -\alpha -1}{\left(1-y\right)}^{\gamma -\beta -1}}{{\left(1-\sigma xy\right)}^{\gamma }}, \end{array}$$ (4)

where

[C(α,β,γ,σ)]-1=B(α,γ-α)B(β,γ-β)3F2(α,β,γ;γ,γ;σ)=B(α,γ-α)B(β,γ-β)2F1(α,β;γ;σ). (5)

Further, re-parameterizing (1) by replacing *α*, *β* and *γ* by *a*, *b* and *a* + *b* + *c*, respectively, a generalized bivariate Kummer-beta pdf is obtained as

$$\begin{array}{c} C(a,b,a+b+c,\sigma ,\lambda_{1},\lambda_{2})\frac{x^{a-1}y^{b-1}{\left(1-x\right)}^{b+c-1}{\left(1-y\right)}^{a+c-1}}{{\left(1-\sigma xy\right)}^{a+b+c}}\;\text{exp}[-(\lambda_{1}x+\lambda_{2}y)]. \end{array}$$ (6)

This pdf for λ_1_ = λ_2_ = 0 and *σ* = 1 is the well known Libby and Novick’s bivariate beta pdf (Libby and Novick [8]). For λ_1_ = λ_2_ = λ and *σ* = 1, it is the central bivariate Kummer-beta Type IV pdf introduced by Jacobs, Bekker and Human [30].

Fig 1 in S1 Appendix shows possible shapes of (1) for selected values of *α*, *β*, *γ*, *σ*, λ_1_, and λ_1_. Expanding (1 − *σxy*)^−*γ*^, *σxy* < 1 in power series in (1), we can write

$$\begin{array}{cc} f(x,y;\alpha ,\beta ,\gamma ,\sigma ,\lambda_{1},\lambda_{2}) & =C(\alpha ,\beta ,\gamma ,\sigma ,\lambda_{1},\lambda_{2})\\ & \;\cdot \sum_{j=0}^{\infty }\frac{{\left(\gamma \right)}_{j}}{K(\alpha +j,\gamma -\alpha ,-\lambda_{1})K(\beta +j,\gamma -\beta ,-\lambda_{2})}\frac{\sigma^{j}}{j!}\\ & \;\cdot f_{\text{KB}}(x;\alpha +j,\gamma -\alpha ,\lambda_{1})f_{\text{KB}}(y;\beta +j,\gamma -\beta ,\lambda_{2}), \end{array}$$ (7)

where *f*_KB_(*v*; *a*, *b*, *ξ*) is the Kummer-beta pdf. Thus, the GBKB pdf is an infinite mixture of the product of Kummer-beta pdfs.

A bivariate distribution with the pdf *f*(*x*, *y*) is said to be totally positive of order 2 (TP_2_) (Balakrishnan and Lai [1]) if

$$\begin{array}{c} f(x_{1},y_{1})f(x_{2},y_{2})\geq f(x_{1},y_{2})f(x_{2},y_{1}) \end{array}$$ (8)

for *x*_1_ ≤ *x*_2_ and *y*_1_ ≤ *y*_2_. In order to prove that the pdf (1) is (TP_2_), we substitute appropriately from (1). We observe that *f*(*x*_1_, *y*_1_)*f*(*x*_2_, *y*_2_) ≥ *f*(*x*_1_, *y*_2_)*f*(*x*_2_, *y*_1_) if and only if (1 − *σx*_1_*y*_2_)(1 − *σx*_2_*y*_1_) ≥ (1 − *σx*_1_*y*_1_)(1 − *σx*_2_*y*_2_), which always holds, Therefore, the generalized bivariate Kummer-beta distribution is TP_2_.

## 3 Marginal and conditional distributions

By integrating *y* in (1), we obtain the marginal pdf of *X* as

$$\begin{array}{cc} f_{X}\left(x\right) & =C(\alpha ,\beta ,\gamma ,\sigma ,\lambda_{1},\lambda_{2})x^{\alpha -1}{\left(1-x\right)}^{\gamma -\alpha -1}\;\text{exp}(-\lambda_{1}x)\\ & \;\cdot \int_{0}^{1}\frac{y^{\beta -1}{\left(1-y\right)}^{\gamma -\beta -1}\;\text{exp}(-\lambda_{2}y)}{{\left(1-\sigma xy\right)}^{\gamma }}\;\text{d}y\\ & =C(\alpha ,\beta ,\gamma ,\sigma ,\lambda_{1},\lambda_{2})B\left(\beta ,\gamma -\beta \right)x^{\alpha -1}{\left(1-x\right)}^{\gamma -\alpha -1}\;\text{exp}(-\lambda_{1}x)\\ & \;\cdot \Phi_{1}(\beta ,\gamma ,\gamma ;\sigma x,-\lambda_{2}), \end{array}$$ (9)

where 0 < *x* < 1. It is interesting to note that the marginal pdf of *X* does not belong to the Kummer-beta family and differs by an additional factor containing the confluent hypergeometric function Φ_1_ of two variables. Likewise, the marginal pdf of *Y*, for 0 < *y* < 1, can be obtained as

$$\begin{array}{cc} f_{Y}\left(y\right) & =C(\alpha ,\beta ,\gamma ,\sigma ,\lambda_{1},\lambda_{2})y^{\beta -1}{\left(1-y\right)}^{\gamma -\beta -1}\;\text{exp}(-\lambda_{2}y)\\ & \;\cdot \int_{0}^{1}\frac{x^{\alpha -1}{\left(1-x\right)}^{\gamma -\alpha -1}\;\text{exp}(-\lambda_{1}x)}{{\left(1-xy\sigma \right)}^{\gamma }}\;\text{d}x\\ & =C(\alpha ,\beta ,\gamma ,\sigma ,\lambda_{1},\lambda_{2})B\left(\alpha ,\gamma -\alpha \right)y^{\beta -1}{\left(1-y\right)}^{\gamma -\beta -1}\;\text{exp}(-\lambda_{2}y)\\ & \;\cdot \Phi_{1}(\alpha ,\gamma ,\gamma ;\sigma y,-\lambda_{1}). \end{array}$$ (10)

Fig 2 in S1 Appendix plots the marginal pdf of *X* for selected values of the parameters. It can be checked that the marginal pdf of *X* is more flexible than existing (for example, Kummer-beta distribution) ones and includes a wide variety of shapes.

From the joint pdf (1) and the marginal pdf of *X* given in (9), the conditional pdf of *Y* given *X* = *x* can be derived as

$$\begin{array}{c} f\left(y|x\right)=\frac{y^{\beta -1}{\left(1-y\right)}^{\gamma -\beta -1}\;\text{exp}(-\lambda_{2}y){\left(1-\sigma xy\right)}^{-\gamma }}{B\left(\beta ,\gamma -\beta \right)\Phi_{1}(\beta ,\gamma ,\gamma ;\sigma x,-\lambda_{2})}. \end{array}$$ (11)

Also, the conditional pdf of *X* given *Y* = *y* is

$$\begin{array}{c} f\left(x|y\right)=\frac{x^{\alpha -1}{\left(1-x\right)}^{\gamma -\alpha -1}\;\text{exp}(-\lambda_{1}x){\left(1-\sigma xy\right)}^{-\gamma }}{B\left(\alpha ,\gamma -\alpha \right)\Phi_{1}(\alpha ,\gamma ,\gamma ;\sigma y,-\lambda_{1})}. \end{array}$$ (12)

## 4 Moments

By definition, the (*r*, *s*)th joint moment can be derived as

$$\begin{array}{cc} E(X^{r}Y^{s}) & =C(\alpha ,\beta ,\gamma ,\sigma ,\lambda_{1},\lambda_{2})\int_{0}^{1}\int_{0}^{1}\frac{x^{\alpha +r-1}y^{\beta +s-1}{\left(1-x\right)}^{\gamma -\alpha -1}{\left(1-y\right)}^{\gamma -\beta -1}}{{\left(1-\sigma xy\right)}^{\gamma }}\\ & \;\;\;\;\;\cdot \;\text{exp}[-(\lambda_{1}x+\lambda_{2}y)]\;\text{d}x\;\text{d}y\\ & =C(\alpha ,\beta ,\gamma ,\sigma ,\lambda_{1},\lambda_{2})B\left(\alpha +r,\gamma -\alpha \right)B\left(\beta +s,\gamma -\beta \right)\\ & \;\cdot F_{Z}(\alpha +r,\beta +s,\gamma ;\gamma +r,\gamma +s;\sigma ,-\lambda_{1},-\lambda_{2}), \end{array}$$ (13)

where the last line follows by using equation (9) in S1 Appendix. Simplifying the above expression by using (2), we obtain

$$\begin{array}{c} E(X^{r}Y^{s})=\frac{\Gamma \left(\alpha +r\right)\Gamma \left(\beta +s\right)\Gamma^{2}\left(\gamma \right)}{\Gamma (\gamma +r)\Gamma (\gamma +s)\Gamma (\alpha )\Gamma (\beta )}\frac{F_{Z}(\alpha +r,\beta +s,\gamma ;\gamma +r,\gamma +s;\sigma ,-\lambda_{1},-\lambda_{2})}{F_{Z}(\alpha ,\beta ,\gamma ;\gamma ,\gamma ;\sigma ,-\lambda_{1},-\lambda_{2})}, \end{array}$$ (14)

where *α* + *r* > 0 and *β* + *s* > 0.

Further, substituting appropriately in the above expression, we obtain

$$\begin{array}{c} E(X^{r})=\frac{\Gamma (\alpha +r)\Gamma (\gamma )}{\Gamma (\gamma +r)\Gamma (\alpha )}\frac{F_{Z}(\alpha +r,\beta ,\gamma ;\gamma +r,\gamma ;\sigma ,-\lambda_{1},-\lambda_{2})}{F_{Z}(\alpha ,\beta ,\gamma ;\gamma ,\gamma ;\sigma ,-\lambda_{1},-\lambda_{2})}, \end{array}$$ (15)

$$\begin{array}{c} E(Y^{s})=\frac{\Gamma (\beta +s)\Gamma (\gamma )}{\Gamma (\gamma +s)\Gamma (\beta )}\frac{F_{Z}(\alpha ,\beta +s,\gamma ;\gamma ,\gamma +s;\sigma ,-\lambda_{1},-\lambda_{2})}{F_{Z}(\alpha ,\beta ,\gamma ;\gamma ,\gamma ;\sigma ,-\lambda_{1},-\lambda_{2})}, \end{array}$$ (16)

$$\begin{array}{c} E\left(XY\right)=\frac{\alpha \beta }{\gamma^{2}}\frac{F_{Z}(\alpha +1,\beta +1,\gamma ;\gamma +1,\gamma +1;\sigma ,-\lambda_{1},-\lambda_{2})}{F_{Z}(\alpha ,\beta ,\gamma ;\gamma ,\gamma ;\sigma ,-\lambda_{1},-\lambda_{2})}, \end{array}$$ (17)

$$\begin{array}{c} E\left(X\right)=\frac{\alpha }{\gamma }\frac{F_{Z}(\alpha +1,\beta ,\gamma ;\gamma +1,\gamma ;\sigma ,-\lambda_{1},-\lambda_{2})}{F_{Z}(\alpha ,\beta ,\gamma ;\gamma ,\gamma ;\sigma ,-\lambda_{1},-\lambda_{2})}, \end{array}$$ (18)

$$\begin{array}{c} E(X^{2})=\frac{\alpha (\alpha +1)}{\gamma (\gamma +1)}\frac{F_{Z}(\alpha +2,\beta ,\gamma ;\gamma +2,\gamma ;\sigma ,-\lambda_{1},-\lambda_{2})}{F_{Z}(\alpha ,\beta ,\gamma ;\gamma ,\gamma ;\sigma ,-\lambda_{1},-\lambda_{2})}, \end{array}$$ (19)

$$\begin{array}{c} E\left(Y\right)=\frac{\beta }{\gamma }\frac{F_{Z}(\alpha ,\beta +1,\gamma ;\gamma ,\gamma +1;\sigma ,-\lambda_{1},-\lambda_{2})}{F_{Z}(\alpha ,\beta ,\gamma ;\gamma ,\gamma ;\sigma ,-\lambda_{1},-\lambda_{2})}, \end{array}$$ (20)

and

$$\begin{array}{c} E(Y^{2})=\frac{\beta (\beta +1)}{\gamma (\gamma +1)}\frac{F_{Z}(\alpha ,\beta +2,\gamma ;\gamma ,\gamma +2;\sigma ,-\lambda_{1},-\lambda_{2})}{F_{Z}(\alpha ,\beta ,\gamma ;\gamma ,\gamma ;\sigma ,-\lambda_{1},-\lambda_{2})}. \end{array}$$ (21)

The conditional moments are given by

$$\begin{array}{cc} E(X^{r}|Y) & =\int_{0}^{1}x^{r}f\left(x|y\right)\;\text{d}x\\ & =\frac{B\left(\alpha +r,\gamma -\alpha \right)\Phi_{1}(\alpha +r,\gamma ,\gamma +r;\sigma y,-\lambda_{1})}{B\left(\alpha ,\gamma -\alpha \right)\Phi_{1}(\alpha ,\gamma ,\gamma ;\sigma y,-\lambda_{1})} \end{array}$$ (22)

and

$$\begin{array}{cc} E(Y^{s}|X) & =\int_{0}^{1}y^{s}f\left(y|x\right)\;\text{d}y\\ & =\frac{B\left(\beta +s,\gamma -\beta \right)\Phi_{1}(\beta +s,\gamma ,\gamma +s;\sigma x,-\lambda_{2})}{B\left(\beta ,\gamma -\beta \right)\Phi_{1}(\beta ,\gamma ,\gamma ;\sigma x,-\lambda_{2})}. \end{array}$$ (23)

From (1), we can easily see that

$$\begin{array}{c} E[{\left(\frac{XY}{1-\sigma XY}\right)}^{r}]=\frac{C(\alpha ,\beta ,\gamma ,\sigma ,\lambda_{1},\lambda_{2})}{C(\alpha +r,\beta +r,\gamma +r,\sigma ,\lambda_{1},\lambda_{2})} \end{array}$$ (24)

which simplifies to

$$\begin{array}{cc} E[{\left(\frac{XY}{1-\sigma XY}\right)}^{r}] & =\frac{\Gamma \left(\alpha +r\right)\Gamma \left(\beta +r\right)\Gamma^{2}\left(\gamma \right)}{\Gamma^{2}\left(\gamma +r\right)\Gamma \left(\alpha \right)\Gamma \left(\beta \right)}\\ & \;\cdot \frac{F_{Z}(\alpha +r,\beta +r,\gamma +r;\gamma +r,\gamma +r;\sigma ,-\lambda_{1},-\lambda_{2})}{F_{Z}(\alpha ,\beta ,\gamma ;\gamma ,\gamma ;\sigma ,-\lambda_{1},-\lambda_{2})}. \end{array}$$ (25)

The joint moment generating function of (*X*, *Y*) can be given by

$$\begin{array}{cc} M_{X,Y}(t_{1},t_{2}) & =E[\;\text{exp}(t_{1}X+t_{2}Y)]\\ & =C(\alpha ,\beta ,\gamma ,\sigma ,\lambda_{1},\lambda_{2})\int_{0}^{1}\int_{0}^{1}\frac{x^{\alpha -1}y^{\beta -1}{\left(1-x\right)}^{\gamma -\alpha -1}{\left(1-y\right)}^{\gamma -\beta -1}}{{\left(1-\sigma xy\right)}^{\gamma }}\\ & \;\cdot \;\text{exp}[t_{1}x+t_{2}y-(\lambda_{1}x+\lambda_{2}y)]\;\text{d}x\;\text{d}y\\ & =\frac{C(\alpha ,\beta ,\gamma ,\sigma ,\lambda_{1},\lambda_{2})}{C(\alpha ,\beta ,\gamma ,\sigma ,\lambda_{1}-t_{1},\lambda_{2}-t_{2})} \end{array}$$ (26)

which simplifies to

$$\begin{array}{c} M_{X,Y}(t_{1},t_{2})=\frac{F_{Z}(\alpha ,\beta ,\gamma ;\gamma ,\gamma ;\sigma ,t_{1}-\lambda_{1},t_{2}-\lambda_{2})}{F_{Z}(\alpha ,\beta ,\gamma ;\gamma ,\gamma ;\sigma ,-\lambda_{1},-\lambda_{2})}. \end{array}$$ (27)

## 5 Entropies

In this section, we derive expressions for Rényi and Shannon entropies for the bivariate beta distribution defined in Section 2.

Let (X,B,P) be a probability space. Consider a pdf *f* associated with P, dominated by a *σ*−finite measure *μ* on X. The well-known Shannon entropy, denoted by *H*_*SH*_(*f*), is defined by

$$\begin{array}{c} H_{SH}\left(f\right)=-\int_{X}f\left(x\right)\;\text{ln}\;f\left(x\right)\;d\mu . \end{array}$$ (28)

One of the main extensions of Shannon entropy is due to Rényi [31]. Rényi entropy, denoted by *H*_*R*_(*η*, *f*), is

$$\begin{array}{c} H_{R}\left(\eta ,f\right)=\frac{\;\text{ln}\;G(\eta )}{1-\eta } \end{array}$$ (29)

for *η* > 0 and *η* ≠ 1, where

$$\begin{array}{c} G\left(\eta \right)=\int_{X}f^{\eta }\;d\mu . \end{array}$$ (30)

The parameter *η* introduced in *H*_*R*_(*η*, *f*) is used to describe the complex behavior in probability models and the associated process under study. Rényi entropy is monotonically decreasing in *η*, while Shannon entropy (28) is obtained from (29) for *η* ↑ 1. For details, see Nadarajah and Zografos [32], Zografos and Nadarajah [33]. First, we give the following lemma useful in deriving these entropies.

**Lemma 1** *Let g*(*α*, *β*, *γ*, *σ*, λ_1_, λ_2_) = lim_*η* → 1_*h*(*η*), *where*

$$\begin{array}{c} h\left(\eta \right)=\frac{d}{d\eta }F_{Z}(\eta \left(\alpha -1\right)+1,\eta \left(\beta -1\right)+1,\eta \gamma ;\eta \left(\gamma -2\right)+2,\eta \left(\gamma -2\right)+2;\sigma ,-\eta \lambda_{1},-\eta \lambda_{2}). \end{array}$$ (31)

*Then*,

$$\begin{array}{cc} g(\alpha ,\beta ,\gamma ,\sigma ,\lambda_{1},\lambda_{2}) & =\frac{\Gamma (\gamma )}{\Gamma (\alpha )\Gamma (\beta )}\sum_{i,j,k=0}^{\infty }\frac{\Gamma (\alpha +i+j)\Gamma (\beta +i+k)\Gamma (\gamma +i)}{\Gamma (\gamma +i+j)\Gamma (\gamma +i+k)}\\ & \;\frac{\sigma^{i}{\left(-\lambda_{1}\right)}^{j}{\left(-\lambda_{2}\right)}^{k}}{i!\;j!\;k!}[j+k+2\left(\gamma -2\right)\psi \left(\gamma \right)-\left(\alpha -1\right)\psi \left(\alpha \right)\\ & \;\;-(\beta -1)\psi (\beta )-\gamma \psi (\gamma )+(\alpha -1)\psi (\alpha +i+j)\\ & \;\;+(\beta -1)\psi (\beta +i+k)+\gamma \psi (\gamma +i)\\ & \;\;-(\gamma -2)\psi (\gamma +i+j)-(\gamma -2)\psi (\gamma +i+k)], \end{array}$$ (32)

*where* $\psi \left(z\right)=\frac{\Gamma^{\prime }\left(z\right)}{\Gamma (z)}$ *is the digamma function.*

**Proof** Expanding *F*_*Z*_ in series form, we can write

$$\begin{array}{cc} h(\eta ) & =\frac{d}{d\eta }\sum_{i,j,k=0}^{\infty }\Delta_{i,j,k}\left(\eta \right)\frac{\sigma^{i}{\left(-\eta \lambda_{1}\right)}^{j}{\left(-\eta \lambda_{2}\right)}^{k}}{i!\;j!\;k!}\\ & =\sum_{i,j,k=0}^{\infty }[\frac{d}{d\eta }\Delta_{i,j,k}\left(\eta \right)]\frac{\sigma^{i}{\left(-\lambda_{1}\right)}^{j}{\left(-\lambda_{2}\right)}^{k}}{i!\;j!\;k!}, \end{array}$$ (33)

where

$$\begin{array}{cc} \Delta_{i,j,k}\left(\eta \right) & =\frac{\Gamma^{2}[\eta (\gamma -2)+2]}{\Gamma [\eta (\alpha -1)+1]\Gamma [\eta (\beta -1)+1]\Gamma \left(\eta \gamma \right)}\\ & \;\cdot \frac{\Gamma [\eta (\alpha -1)+1+i+j]\Gamma [\eta (\beta -1)+1+i+k]\Gamma \left(\eta \gamma +i\right)}{\Gamma [\eta (\gamma -2)+2+i+j]\Gamma [\eta (\gamma -2)+2+i+k]}\eta^{j+k}. \end{array}$$ (34)

Now, differentiating the logarithm of Δ_*i*,*j*, *k*_(*η*) with respect to *η*, we obtain

$$\begin{array}{cc} \frac{d}{d\eta }\Delta_{i,j,k}\left(\eta \right) & =\Delta_{i,j,k}\left(\eta \right)[\frac{j+k}{\eta }+2\left(\gamma -2\right)\psi (\eta (\gamma -2)+2)-\left(\alpha -1\right)\psi (\eta (\alpha -1)+1)\\ & \;-\left(\beta -1\right)\psi (\eta (\beta -1)+1)-\gamma \psi \left(\eta \gamma \right)+\left(\alpha -1\right)\psi (\eta (\alpha -1)+1+i+j)\\ & \;+\left(\beta -1\right)\psi (\eta (\beta -1)+1+i+k)+\gamma \psi \left(\eta \gamma +i\right)\\ & \;-\left(\gamma -2\right)\psi (\eta (\gamma -2)+2+i+j)-\left(\gamma -2\right)\psi (\eta (\gamma -2)+2+i+k)]. \end{array}$$ (35)

Finally, substituting (35) into (33) and taking *η* → 1, we obtain the desired result.

**Theorem 1** *For the generalized bivariate beta distribution defined by the pdf* (1), *Rényi and Shannon entropies are given by*

$$\begin{array}{cc} H_{R}\left(\eta ,f\right) & =\frac{1}{1-\eta }[\eta \;\text{ln}\;C(\alpha ,\beta ,\gamma ,\sigma ,\lambda_{1},\lambda_{2})+\;\text{ln}\;\Gamma [\eta (\alpha -1)+1]+\;\text{ln}\;\Gamma [\eta (\beta -1)+1]\\ & \;+\;\text{ln}\;\Gamma [\eta (\gamma -\alpha -1)+1]+\;\text{ln}\;\Gamma [\eta (\gamma -\beta -1)+1]-2\;\text{ln}\;\Gamma [\eta (\gamma -2)+2]\\ & \;+\;\text{ln}\;F_{Z}(\eta \left(\alpha -1\right)+1,\eta \left(\beta -1\right)+1,\eta \gamma ;\eta \left(\gamma -2\right)+2,\eta \left(\gamma -2\right)+2;\sigma ,-\eta \lambda_{1},-\eta \lambda_{2})] \end{array}$$ (36)

*and*

$$\begin{array}{cc} H_{SH}\left(f\right) & =-\;\text{ln}\;C(\alpha ,\beta ,\gamma ,\sigma ,\lambda_{1},\lambda_{2})-[\left(\alpha -1\right)\psi \left(\alpha \right)+\left(\beta -1\right)\psi \left(\beta \right)\\ & \;+(\gamma -\alpha -1)\psi (\gamma -\alpha )+(\gamma -\beta -1)\psi (\gamma -\beta )-2(\gamma -2)\psi (2\gamma )]\\ & \;-\frac{g(\alpha ,\beta ,\gamma ,\sigma ,\lambda_{1},\lambda_{2})}{F_{Z}(\alpha ,\beta ,\gamma ;\gamma ,\gamma ;\sigma ,-\lambda_{1},-\lambda_{2})}, \end{array}$$ (37)

*respectively.*

**Proof** For *η* > 0 and *η* ≠ 1, using (1), we have

$$\begin{array}{cc} G(\eta ) & =\int_{0}^{1}\int_{0}^{1}f^{\eta }(x_{1},x_{2};\alpha ,\beta ,\gamma ,\sigma ,\lambda_{1},\lambda_{2})\;\text{d}x\;\text{d}y\\ & ={\left[C(\alpha ,\beta ,\gamma ,\sigma ,\lambda_{1},\lambda_{2})\right]}^{\eta }\\ & \;\cdot \int_{0}^{1}\int_{0}^{1}\frac{x^{\eta (\alpha -1)}y^{\eta (\beta -1)}{\left(1-x\right)}^{\eta (\gamma -\alpha -1)}{\left(1-y\right)}^{\eta (\gamma -\beta -1)}}{{\left(1-\sigma xy\right)}^{\eta \gamma }}\;\text{exp}[-\eta (\lambda_{1}x+\lambda_{2}y)]\;\text{d}x\;\text{d}y\\ & ={\left[C(\alpha ,\beta ,\gamma ,\sigma ,\lambda_{1},\lambda_{2})\right]}^{\eta }B(\eta (\alpha -1)+1,\eta (\gamma -\alpha -1)+1)\\ & \;\cdot B(\eta (\beta -1)+1,\eta (\gamma -\beta -1)+1)\\ & \;\cdot F_{Z}(\eta \left(\alpha -1\right)+1,\eta \left(\beta -1\right)+1,\eta \gamma ;\eta \left(\gamma -2\right)+2,\eta \left(\gamma -2\right)+2;\sigma ,-\eta \lambda_{1},-\eta \lambda_{2}), \end{array}$$ (38)

where the last line follows by equation (9) in S1 Appendix. Now, taking logarithm of *G*(*η*) and using (29), we obtain *H*_*R*_(*η*, *f*). Shannon entropy can be obtained from *H*_*R*_(*η*, *f*) by taking *η* ↑ 1 and using L’Hopital’s rule.

## 6 Product and quotient

Distributions of products and ratios of correlated random variables are of interest in many areas of science such as economics, genetics, hydrology, meteorology, nuclear physics, and reliability (see, for instance, Nadarajah and Choi [34] and Nadarajah and Ruiz-Espejo [35]). Several distributional results on products and ratios are available for normal, gamma, exponential, Pareto, Rayleigh and Weibull families of bivariate distributions. However, there is relatively little work of the this kind when the joint distribution is bivariate beta.

In this section, we derive distributions of *XY* and $\frac{X}{Y}$ when *X* and *Y* follow the distribution defined in (1).

**Theorem 3** *Let* (*X*, *Y*) ∼ GBKB(*α*, *β*, *γ*, *σ*, λ_1_, *and P* = *X*_1_*X*_2_. *Then, the pdf of P is*

$$\begin{array}{c} C(\alpha ,\beta ,\gamma ,\sigma ,\lambda_{1},\lambda_{2})\;\text{exp}(-\lambda_{2})\frac{p^{\alpha -1}{\left(1-p\right)}^{2\gamma -\alpha -\beta -1}}{{\left(1-\sigma p\right)}^{\gamma }}\sum_{j=0}^{\infty }\frac{{\left(-\lambda_{1}p\right)}^{j}}{j!}\\ \;\cdot B\left(\gamma -\alpha ,\gamma -\beta \right)\Phi_{1}(\gamma -\beta ,\gamma -\beta +j,2\gamma -\alpha -\beta ;1-p,\lambda_{2}\left(1-p\right)),\;0<p<1. \end{array}$$ (39)

**Proof** Transforming *P* = *XY*, *Y* = *Y* with the Jacobian $J\left(x,y\to z,y\right)=\frac{1}{y}$ in (1), we obtain the joint pdf of *P* and *Y* as

$$\begin{array}{c} C(\alpha ,\beta ,\gamma ,\sigma ,\lambda_{1},\lambda_{2})\frac{p^{\alpha -1}{\left(y-p\right)}^{\gamma -\alpha -1}{\left(1-y\right)}^{\gamma -\beta -1}}{y^{\gamma -\beta }{\left(1-\sigma p\right)}^{\gamma }}\;\text{exp}[-(\lambda_{1}\frac{p}{y}+\lambda_{2}y)], \end{array}$$ (40)

where 0 < *p* < *y* < 1. Integrating this expression with respect to *y*, we can write the pdf of *P* as

$$\begin{array}{c} C(\alpha ,\beta ,\gamma ,\sigma ,\lambda_{1},\lambda_{2})\frac{p^{\alpha -1}}{{\left(1-\sigma p\right)}^{\gamma }}\sum_{j=0}^{\infty }\frac{{\left(-\lambda_{1}p\right)}^{j}}{j!}\\ \;\cdot \int_{p}^{1}\frac{{\left(y-p\right)}^{\gamma -\alpha -1}{\left(1-y\right)}^{\gamma -\beta -1}}{y^{\gamma -\beta +j}}\;\text{exp}(-\lambda_{2}y)\;\text{d}y. \end{array}$$ (41)

Now, substitute $w=\frac{1-y}{1-p}$ and rewrite

$$\begin{array}{c} C(\alpha ,\beta ,\gamma ,\sigma ,\lambda_{1},\lambda_{2})\;\text{exp}(-\lambda_{2})\frac{p^{\alpha -1}{\left(1-p\right)}^{2\gamma -\alpha -\beta -1}}{{\left(1-\sigma p\right)}^{\gamma }}\sum_{j=0}^{\infty }\frac{{\left(-\lambda_{1}p\right)}^{j}}{j!}\\ \;\cdot \int_{0}^{1}\frac{{\left(1-w\right)}^{\gamma -\alpha -1}w^{\gamma -\beta -1}}{{\left[1-(1-p)w\right]}^{\gamma -\beta +j}}\;\text{exp}[\lambda_{2}\left(1-p\right)w]\;dw. \end{array}$$ (42)

Finally, the desired result is obtained by evaluating the integral by using equation (7) in S1 Appendix.

**Theorem 4** *Let* (*X*, *Y*) ∼ GBKB(*α*, *β*, *γ*, *σ*, λ_2_ and $R=\frac{X}{Y}$. *Then, the pdf of R is*

$$\begin{array}{c} C(\alpha ,\beta ,\gamma ,\sigma ,\lambda_{1},\lambda_{2})r^{\alpha -1}\sum_{j=0}^{\infty }{\left(\gamma \right)}_{j}\frac{{\left(\sigma r\right)}^{j}}{j!}B\left(\alpha +\beta +2j,\gamma -\beta \right)\\ \;\cdot \Phi_{1}(\alpha +\beta +2j,1+\alpha -\gamma ,\alpha +\gamma +2j;r,\lambda_{2}+\lambda_{1}r) \end{array}$$ (43)

*for* 0 < *r* ≤ 1 *For r* > 1,

$$\begin{array}{c} C(\alpha ,\beta ,\gamma ,\sigma ,\lambda_{1},\lambda_{2})r^{-\beta -1}\sum_{j=0}^{\infty }{\left(\gamma \right)}_{j}\frac{\sigma^{j}}{r^{j}j!}B\left(\alpha +\beta +2j,\gamma -\alpha \right)\\ \;\cdot \Phi_{1}(\alpha +\beta +2j,1+\beta -\gamma ,\beta +\gamma +2j;\frac{1}{r},\lambda_{1}+\frac{\lambda_{2}}{r}). \end{array}$$ (44)

**Proof** Consider the transformation $R=\frac{X}{Y}$, *Y* = *Y* whose Jacobian is *J*(*x*, *y* → *r*, *y*) = *y*. Using (1), we obtain the joint pdf of *R* and *Y* as

$$\begin{array}{c} C(\alpha ,\beta ,\gamma ,\sigma ,\lambda_{1},\lambda_{2})\frac{r^{\alpha -1}y^{\alpha +\beta -1}{\left(1-ry\right)}^{\gamma -\alpha -1}{\left(1-y\right)}^{\gamma -\beta -1}}{{\left(1-\sigma ry^{2}\right)}^{\gamma }}\;\text{exp}[-(\lambda_{1}r+\lambda_{2})y], \end{array}$$ (45)

where 0 < *y* < 1 for 0 < *r* ≤ 1, and $0<y<\frac{1}{r}$ for *r* > 1. For 0 < *r* ≤ 1, the marginal pdf of *R* can be obtained by integrating (45) over 0 < *y* < 1, yielding

$$\begin{array}{c} C(\alpha ,\beta ,\gamma ,\sigma ,\lambda_{1},\lambda_{2})r^{\alpha -1}\int_{0}^{1}\frac{y^{\alpha +\beta -1}{\left(1-ry\right)}^{\gamma -\alpha -1}{\left(1-y\right)}^{\gamma -\beta -1}}{{\left(1-\sigma ry^{2}\right)}^{\gamma }}\;\text{exp}[-(\lambda_{1}r+\lambda_{2})y]\;\text{d}y\\ =C(\alpha ,\beta ,\gamma ,\sigma ,\lambda_{1},\lambda_{2})r^{\alpha -1}\;\sum_{j=0}^{\infty }\;{\left(\gamma \right)}_{j}\frac{{\left(\sigma r\right)}^{j}}{j!}\;\int_{0}^{1}\frac{y^{\alpha +\beta +2j-1}{\left(1-y\right)}^{\gamma -\beta -1}}{{\left(1-ry\right)}^{\alpha +1-\gamma }}\;\text{exp}[-(\lambda_{1}r+\lambda_{2})y]\;\text{d}y, \end{array}$$ (46)

where the last line follows by expanding (1 − *σry*^2^)^−*γ*^ in power series. Now, evaluating the integral using equation (7) in S1 Appendix gives the desired result. For *r* > 1, the pdf of *R* can be written as

$$\begin{array}{c} C(\alpha ,\beta ,\gamma ,\sigma ,\lambda_{1},\lambda_{2})r^{\alpha -1}\int_{0}^{\frac{1}{r}}\frac{y^{\alpha +\beta -1}{\left(1-ry\right)}^{\gamma -\alpha -1}{\left(1-y\right)}^{\gamma -\beta -1}}{{\left(1-\sigma ry^{2}\right)}^{\gamma }}\;\text{exp}[-(\lambda_{1}r+\lambda_{2})y]\;\text{d}y\\ =C(\alpha ,\beta ,\gamma ,\sigma ,\lambda_{1},\lambda_{2})\\ \;\cdot r^{-\beta -1}\int_{0}^{1}\frac{w^{\alpha +\beta -1}{\left(1-w\right)}^{\gamma -\alpha -1}{\left(1-\frac{w}{r}\right)}^{\gamma -\beta -1}}{{\left(1-\frac{\sigma w^{2}}{r}\right)}^{\gamma }}\;\text{exp}[-(\lambda_{1}+\frac{\lambda_{2}}{r})w]dw\\ =C(\alpha ,\beta ,\gamma ,\sigma ,\lambda_{1},\lambda_{2})r^{-\beta -1}\sum_{j=0}^{\infty }{\left(\gamma \right)}_{j}\frac{\sigma^{j}}{r^{j}j!}\\ \;\cdot \int_{0}^{1}\frac{w^{\alpha +\beta +2j-1}{\left(1-w\right)}^{\gamma -\alpha -1}}{{\left(1-\frac{w}{r}\right)}^{\beta +1-\gamma }}\;\text{exp}[-(\lambda_{1}+\frac{\lambda_{2}}{r})w]dw. \end{array}$$ (47)

Now, evaluating this integral using equation (7) in S1 Appendix yields the desired result.

## 7 Estimation

Let (*X*_1_, *Y*_1_), …, (*X*_*n*_, *Y*_*n*_) be a random sample from $GBKB(\alpha ,\beta ,\gamma ,\sigma ,\lambda_{1},\lambda_{2})$. The log-likelihood function, denoted by *l*(*α*, *β*, *γ*, *σ*, λ_1_, λ_2_), is

$$\begin{array}{c} l(\alpha ,\beta ,\gamma ,\sigma ,\lambda_{1},\lambda_{2})=n\;\text{ln}\;C(\alpha ,\beta ,\gamma ,\sigma ,\lambda_{1},\lambda_{2})+\left(\alpha -1\right)\sum_{i=1}^{n}\;\text{ln}\;x_{i}+\left(\beta -1\right)\sum_{i=1}^{n}\;\text{ln}\;y_{i}\\ \;+\left(\gamma -\alpha -1\right)\sum_{i=1}^{n}\;\text{ln}\;(1-x_{i})+\left(\gamma -\beta -1\right)\sum_{i=1}^{n}\;\text{ln}\;(1-y_{i})\\ \;-\lambda_{1}\sum_{i=1}^{n}x_{i}-\lambda_{2}\sum_{i=1}^{n}y_{i}-\gamma \sum_{i=1}^{n}\;\text{ln}\;(1-\sigma x_{i}y_{i}). \end{array}$$ (48)

By differentiating *l*(*α*, *β*, *γ*, *σ*, λ_1_, λ_2_) with respect to *α*, *β*, *γ*, *σ*, λ_1_, λ_2_ and equating the resulting expressions to zero, six likelihood equations can be derived as

$$\begin{array}{c} \frac{\partial l(\alpha ,\beta ,\gamma ,\sigma ,\lambda_{1},\lambda_{2})}{\partial \alpha }=\sum_{i=1}^{n}\;\text{ln}\;x_{i}-\sum_{i=1}^{n}\;\text{ln}\;(1-x_{i})+n\frac{\partial \;\text{ln}\;C(\alpha ,\beta ,\gamma ,\sigma ,\lambda_{1},\lambda_{2})}{\partial \alpha }=0, \end{array}$$ (49)

$$\begin{array}{c} \frac{\partial l(\alpha ,\beta ,\gamma ,\sigma ,\lambda_{1},\lambda_{2})}{\partial \beta }=\sum_{i=1}^{n}\;\text{ln}\;y_{i}-\sum_{i=1}^{n}\;\text{ln}\;(1-y_{i})+n\frac{\partial \;\text{ln}\;C(\alpha ,\beta ,\gamma ,\sigma ,\lambda_{1},\lambda_{2})}{\partial \beta }=0, \end{array}$$ (50)

$$\begin{array}{c} \frac{\partial l(\alpha ,\beta ,\gamma ,\sigma ,\lambda_{1},\lambda_{2})}{\partial \gamma }=\sum_{i=1}^{n}\;\text{ln}\;(1-x_{i})+\sum_{i=1}^{n}\;\text{ln}\;(1-y_{i})-\sum_{i=1}^{n}\;\text{ln}\;(1-x_{i}y_{i}\sigma )\\ \;+n\frac{\partial \;\text{ln}\;C(\alpha ,\beta ,\gamma ,\sigma ,\lambda_{1},\lambda_{2})}{\partial \gamma }=0, \end{array}$$ (51)

$$\begin{array}{c} \frac{\partial l(\alpha ,\beta ,\gamma ,\sigma ,\lambda_{1},\lambda_{2})}{\partial \sigma }=-\gamma \sum_{i=1}^{n}\frac{-x_{i}y_{i}}{1-x_{i}y_{i}\sigma }+n\frac{\partial \;\text{ln}\;C(\alpha ,\beta ,\gamma ,\sigma ,\lambda_{1},\lambda_{2})}{\partial \sigma }=0, \end{array}$$ (52)

$$\begin{array}{c} \frac{\partial l(\alpha ,\beta ,\gamma ,\sigma ,\lambda_{1},\lambda_{2})}{\partial \lambda_{1}}=-\sum_{i=1}^{n}x_{i}+n\frac{\partial \;\text{ln}\;C(\alpha ,\beta ,\gamma ,\sigma ,\lambda_{1},\lambda_{2})}{\partial \lambda_{1}}=0, \end{array}$$ (53)

$$\begin{array}{c} \frac{\partial l(\alpha ,\beta ,\gamma ,\sigma ,\lambda_{1},\lambda_{2})}{\partial \lambda_{2}}=-\sum_{i=1}^{n}y_{i}+n\frac{\partial \;\text{ln}\;C(\alpha ,\beta ,\gamma ,\sigma ,\lambda_{1},\lambda_{2})}{\partial \lambda_{2}}=0. \end{array}$$ (54)

The derivatives of the normalizing constant *C*(*α*, *β*, *γ*, *σ*, λ_1_, λ_2_) with respect to the six parameters appear difficult. Thereby, usable solutions of the set of equations can not be obtained and numerical solutions for the maximum likelihood estimates appear to be the only plausible alternatives.

## 8 Simulation study

According to large sample theory, the maximum likelihood estimators of *α*, *β*, *γ*, *σ*, λ_1_, λ_2_ should have zero biases and zero mean squared errors as the sample size approaches infinity. In this section, we perform a simulation study to check the finite sample performance of the maximum likelihood estimators.

Samples of size *n* = 30, 250, 500, 1000 were generated from (1) for selected values of parameters. MCMC methods (Gibbs Metropolis, Markov Chain Monte Carlo Metropolis, Metropolis, Metropolis Gaussian, random walk Metropolis and Metropolis-Hastings) were used with the aid of R packages MCMC, MCMCpack, gibbs.met, LearnBayes, MHadaptive, MetroHastings and walkMetropolis.

We considered three sets of parameter values for simulation as follows:

$$\begin{array}{c} \alpha =2,\beta =2,\gamma =4,\sigma =0.5,\lambda_{1}=1,\lambda_{2}=1, \end{array}$$ (55)

$$\begin{array}{c} \alpha =2,\beta =2,\gamma =6,\sigma =0.5,\lambda_{1}=2,\lambda_{2}=2, \end{array}$$ (56)

and

$$\begin{array}{c} \alpha =2,\beta =2,\gamma =4,\sigma =0.5,\lambda_{1}=1.5,\lambda_{2}=1.5. \end{array}$$ (57)

The results were similar for other choices of parameters. The maximum likelihood estimates of *α*, *β*, *γ*, *σ*, λ_1_, λ_2_ were computed based on numerical procedures. These procedures were repeated one hundred times to give the average of biases (Ab) and the average of mean squared errors (MSE). By comparing these results, we observed that the Gibbs sampling method provided better results. Therefore, the output of the Gibbs method is presented in Tables 1–6 and Figs 3 to 7 in S1 Appendix. The maximum likelihood estimates and correlation coefficients are reported in Tables 1–3. The average of biases and the mean squared errors of all estimators are reported in Tables 4–6. The biases always appear close enough to 0 and the mean squared errors of all estimators always decrease with increasing *n*. Figs 3 and 4 in S1 Appendix show scatter plots of the simulated data for *n* = 30 and *n* = 1000. Fig 5 in S1 Appendix shows pairs style of Gibbs sampling method for *α* = 2, *β* = 2, *γ* = 4, *σ* = 0.5, λ_1_ = 1, λ_2_ = 1, *n* = 1000. Fig 6 in S1 Appendix shows histogram of the simulated data and marginal pdf for *α* = 2, *β* = 2, *γ* = 4, *σ* = 0.5, λ_1_ = 1, λ_2_ = 1, *n* = 1000. Fig 7 in S1 Appendix shows the correlation between *X* and *Y* for *α* = 2, *β* = 2, *γ* = 4, *σ* = 0.5, λ_1_ = 1, λ_2_ = 1, *n* = 1000. All figures and tables demonstrate that the simulation is satisfactory.

**Table 1:** Maximum likelihood estimates based on simulated data for *α* = 2, *β* = 2, *γ* = 4, *σ* = 0.5, λ_1_ = 1, λ_2_ = 1.

*n*	$\hat{\alpha }$	$\hat{\beta }$	$\hat{\gamma }$	$\hat{\sigma }$	${\hat{\lambda }}_{1}$	${\hat{\lambda }}_{2}$
30	1.973836	2.015562	4.045190	0.491698	1.275161	1.473856
250	1.973836	2.015562	4.045190	0.491698	1.275161	1.473856
500	2.001270	1.980189	4.016316	0.489125	0.958321	0.844904
1000	1.989758	1.979811	4.001838	0.492810	0.926023	0.879106

**Table 2:** Maximum likelihood estimates based on simulated data for *α* = 2, *β* = 2, *γ* = 6, *σ* = 0.5, λ_1_ = 2, λ_2_ = 2.

*n*	$\hat{\alpha }$	$\hat{\beta }$	$\hat{\gamma }$	$\hat{\sigma }$	${\hat{\lambda }}_{1}$	${\hat{\lambda }}_{2}$
30	1.973836	2.015562	4.045110	0.491698	1.275161	1.473856
250	1.975575	2.011879	6.465107	0.460187	1.2218307	1.260903
500	1.985697	2.028305	6.1880215	0.466124	1.644022	1.785429
1000	2.006863	2.024450	6.094308	0.486453	1.893386	1.940943

**Table 3:** Maximum likelihood estimates based on simulated data for *α* = 2, *β* = 2, *γ* = 4, *σ* = 0.5, λ_1_ = 1.5, λ_2_ = 1.5.

*n*	$\hat{\alpha }$	$\hat{\beta }$	$\hat{\gamma }$	$\hat{\sigma }$	${\hat{\lambda }}_{1}$	${\hat{\lambda }}_{2}$
30	2.006863	2.024449	6.094308	0.486453	1.893386	1.940942
250	1.988866	2.001390	4.061478	0.490607	1.370597	1.373026
500	1.973836	2.015562	4.045190	0.491698	1.275161	1.473856
1000	1.973836	2.015562	4.045190	0.491698	1.275161	1.473856

**Table 4:** Biases and MSEs of maximum likelihood estimates based on simulated data for *α* = 2, *β* = 2, *γ* = 4, *σ* = 0.5, λ_1_ = 1, λ_2_ = 1.

*n*	$Ab(\hat{\alpha })$	$Ab(\hat{\beta })$	$Ab(\hat{\gamma })$	$Ab(\hat{\sigma })$	$Ab({\hat{\lambda }}_{1})$	$Ab({\hat{\lambda }}_{2})$	$MSE(\hat{\alpha })$	$MSE(\hat{\beta })$	$MSE(\hat{\gamma })$	$MSE(\hat{\sigma })$	$MSE({\hat{\lambda }}_{1})$	$MSE({\hat{\lambda }}_{2})$
30	-0.026164	0.015562	0.045190	-0.008302	-0.224839	-0.026144	0.057689	0.054870	0.025016	0.014351	1.634038	1.653448
250	-0.026164	0.015562	0.045190	-0.008302	-0.224839	-0.026144	0.057689	0.054870	0.025016	0.014351	1.634038	1.653448
500	0.001270	-0.019812	0.016316	-0.010875	-0.041680	-0.155096	0.052173	0.047800	0.026537	0.011502	1.395474	1.150310
1000	-0.010242	-0.020189	0.001838	-0.007191	-0.073977	-0.120894	0.0263411	0.028684	0.012473	0.006967	0.586353	0.747648

**Table 5:** Biases and MSEs of maximum likelihood estimates based on simulated data for *α* = 2, *β* = 2, *γ* = 6, *σ* = 0.5, λ_1_ = 2, λ_2_ = 2.

*n*	$Ab(\hat{\alpha })$	$Ab(\hat{\beta })$	$Ab(\hat{\gamma })$	$Ab(\hat{\sigma })$	$Ab({\hat{\lambda }}_{1})$	$Ab({\hat{\lambda }}_{2})$	$MSE(\hat{\alpha })$	$MSE(\hat{\beta })$	$MSE(\hat{\gamma })$	$MSE(\hat{\sigma })$	$MSE({\hat{\lambda }}_{1})$	$MSE({\hat{\lambda }}_{2})$
30	-0.026164	0.015562	0.045190	-0.008302	-0.224839	-0.026144	0.057689	0.054870	3.844256	0.014351	2.083716	1.705735
250	-0.024425	0.011879	0.465107	-0.039813	-0.778170	-0.739097	0.067049	0.0788361	1.323920	0.068091	8.123404	7.477096
500	-0.014303	0.028304	0.188021	-0.033876	-0.355978	-0.214571	0.030900	0.035499	0.369252	0.0360234	2.975318	3.152226
1000	0.006863	0.024449	0.094308	-0.013547	-0.106614	-0.059057	0.018835	0.024330	0.176120	0.018613	1.248495	1.769518

**Table 6:** Biases and MSEs of maximum likelihood estimates based on simulated data for *α* = 2, *β* = 2, *γ* = 4, *σ* = 0.5, λ_1_ = 1.5, λ_2_ = 1.5.

*n*	$Ab(\hat{\alpha })$	$Ab(\hat{\beta })$	$Ab(\hat{\gamma })$	$Ab(\hat{\sigma })$	$Ab({\hat{\lambda }}_{1})$	$Ab({\hat{\lambda }}_{2})$	$MSE(\hat{\alpha })$	$MSE(\hat{\beta })$	$MSE(\hat{\gamma })$	$MSE(\hat{\sigma })$	$MSE({\hat{\lambda }}_{1})$	$MSE({\hat{\lambda }}_{2})$
30	0.006863	0.024449	0.094308	-0.013547	-0.106614	-0.059057	0.018835	0.024330	4.553353	0.018613	1.391882	1.960461
250	-0.011134	0.001390	0.061478	-0.009392	-0.129403	-0.126974	0.080879	0.100175	0.053638	0.021369	2.148867	2.632752
500	-0.026164	0.015562	0.045190	-0.008302	-0.224839	-0.026144	0.057689	0.054870	0.025016	0.014351	1.608877	1.429591
1000	-0.026164	0.015562	0.045190	-0.008302	-0.224839	-0.026144	0.057689	0.054870	0.025016	0.014351	1.608877	1.429591

## 9 Conclusions

In this paper, we have proposed a new form of bivariate Kummer-beta distribution with six parameters extending several widely known bivariate beta distributions. The proposed distribution is more flexible than existing ones and includes a wide variety of shapes. Several properties of this distribution have been studied. Since bivariate beta distributions occur frequently in many practical situations, the generalized model presented in this paper will certainly attract attention of users for its applications in different disciplines.

The limitations of the proposed distribution include the normalizing constant (1) not being elementary, the marginal distributions corresponding to (1) not belonging to the Kummer-beta family and maximum likelihood estimation involving solving of six highly non-liner equations. Future work is to consider other means of estimation, including Bayesian estimation, minimum distance estimation, probability weighted moments estimation, least squares estimation, weighted least squares estimation, percentiles estimation, moments estimation, *L* moments estimation and trimmed *L* moments estimation.

## Supporting information

S1 Appendix Special functions and figures. (PDF)

S2 Appendix R codes. (PDF)
