# Supplementary material for: Generalized bivariate Kummer-beta distribution with marginals defined on the unit interval
Source: PLoS One. 2024 Oct 28;19(10):e0311888. doi: 10.1371/journal.pone.0311888 (PMC11516163; doi:10.1371/journal.pone.0311888)
Supplement: S1 Appendix — (PDF) [file pone.0311888.s001.pdf]

Appendix A

The Pochammer symbol $(a)_n$ is defined by
$(a)_n = a(a+1)\cdots(a+n-1) = (a)_{n-1}(a+n-1)$ for $n = 1, 2, \ldots,$ and $(a)_0 = 1$. The generalized hypergeometric function of scalar argument is defined by

$$ {}_pF_q\left(a_1, \ldots, a_p; b_1, \ldots, b_q; z\right) = \sum_{k=0}^{\infty} \frac{(a_1)_k \cdots (a_p)_k}{(b_1)_k \cdots (b_q)_k} \frac{z^k}{k!}, \tag{A.1} $$

where a_i, $i = 1, \ldots, p$, b_j, $j = 1, \ldots, q$ are complex numbers with suitable restrictions and z is a complex variable. Conditions for the convergence of the series in (A.1) are available in the literature, see Luke [1]. It is easy to see from (A.1) that

$$ {}_1F_1(a; c; z) = \sum_{k=0}^{\infty} \frac{(a)_k}{(c)_k} \frac{z^k}{k!}, \tag{A.2} $$

and

$$ {}_2F_1(a, b; c; z) = \sum_{k=0}^{\infty} \frac{(a)_k (b)_k}{(c)_k} \frac{z^k}{k!}, \quad |z| < 1. \tag{A.3} $$

The integral representations of the confluent hypergeometric function (first kind) and the Gauss hypergeometric function are

$$ {}_1F_1(a; c; z) = \frac{\Gamma(c)}{\Gamma(a)\Gamma(c-a)} \int_0^1 t^{a-1}(1-t)^{c-a-1} \exp(zt) \, \mathrm{d}t, \ \mathrm{Re}(c) > \mathrm{Re}(a) > 0, \tag{A.4} $$

and

$$ \begin{gathered} {}_2F_1(a, b; c; z) = \frac{\Gamma(c)}{\Gamma(a)\Gamma(c-a)} \int_0^1 t^{a-1}(1-t)^{c-a-1}(1-zt)^{-b} \, \mathrm{d}t, \\ \mathrm{Re}(c) > \mathrm{Re}(a) > 0, \ |\arg(1-z)| < \pi, \end{gathered} \tag{A.5} $$

respectively. Note that the series expansions for ${}_1F_1$ and ${}_2F_1$ given in (A.2) and (A.3), respectively, can be obtained by expanding $\exp(zt)$ and $(1-zt)^{-b}$, $|zt| < 1$ in (A.4) and (A.5) and integrating t.

The confluent hypergeometric function ${}_1F_1(a; c; z)$ satisfies Kummer's relation

$$ {}_1F_1(a; c; -z) = \exp(-z) {}_1F_1(c-a; c; z). \tag{A.6} $$

For properties and further results on these functions the reader is referred to Luke [1].

Next, we define Humbert's confluent hypergeometric function Φ_1 of x and y and give its series expansions. Following Prudnikov et al. [2, equation 7.2.4(48)], Φ_1 is defined as

$$ \Phi_1(a, b, c; x, y) = \frac{1}{B(a, c-a)} \int_0^1 t^{a-1}(1-t)^{c-a-1}(1-xt)^{-b} \exp(yt) \, \mathrm{d}t, \tag{A.7} $$

where $a > 0$, $c - a > 0$, $|x| < 1$ and $-\infty < y < \infty$. By expanding $(1-xt)^{-b}$, $|tx| < 1$ in power series and integrating t by using the integral representation of the confluent hypergeometric function, the integral can be written as

$$ \begin{aligned} \Phi_1(a, b; c; x, y) &= \sum_{i=0}^{\infty} \frac{(a)_i (b)_i}{(c)_i} \frac{x^i}{i!} \ {}_1F_1(a+i; c+i; y) \\ &= \sum_{i=0}^{\infty} \sum_{j=0}^{\infty} \frac{(a)_{i+j} (b)_i}{(c)_{i+j}} \frac{x^i y^j}{i! \ j!}, \end{aligned} \tag{A.8} $$

where the last line follows by using the series expansion of ${}_1F_1$.

Next, we define a triple hypergeometric function F_Z as

$$F_Z\left(a_1, a_2, b; c_1, c_2; x, y, z\right) = \frac{1}{B\left(a_1, c_1 - a_1\right) B\left(a_2, c_2 - a_2\right)} \cdot \int_0^1 \int_0^1 \frac{s^{a_1-1}(1-s)^{c_1-a_1-1} t^{a_2-1}(1-t)^{c_2-a_2-1} \exp(ys+zt)}{(1-xst)^b}\, \mathrm{d}s\, \mathrm{d}t, \tag{A.9}$$

where $a_1 > 0$, $a_2 > 0$, $c_1 - a_1 > 0$, $c_2 - a_2 > 0$, $|x| < 1$ and $-\infty < y, z < \infty$. Integrating this expression with respect to t by using (A.7), another form of $F_Z\left(a_1, a_2, b; c_1, c_2; x, y, z\right)$ can be derived as

$$F_Z\left(a_1, a_2, b; c_1, c_2; x, y, z\right) = \frac{1}{B\left(a_1, c_1 - a_1\right)} \int_0^1 s^{a_1-1}(1-s)^{c_1-a_1-1} \exp(ys) \Phi_1\left(a_2, b; c_2; xs, y\right)\, \mathrm{d}s.$$

Expanding $\exp(zt)$ in power series and integrating t in (A.9), we obtain

$$F_Z\left(a_1, a_2, b; c_1, c_2; x, y, z\right) = \frac{1}{B\left(a_1, c_1 - a_1\right)} \sum_{j=0}^{\infty} \frac{\left(a_2\right)_i\ z^j}{\left(c_2\right)_j\ j!} \int_0^1 s^{a_1-1}(1-s)^{c_1-a_1-1} \exp(ys)\ {}_2F_1\left(a_2 + j, b; c_2 + j, xs\right)\, \mathrm{d}s.$$

By expanding $(1-xst)^{-b}$ in power series and integrating s and t by using the integral representation of the confluent hypergeometric function, the integral in (A.9) can be evaluated as

$$\begin{aligned} F_Z\left(a_1, a_2, b; c_1, c_2; x, y, z\right) &= \sum_{i=0}^{\infty} \frac{(b)_i \left(a_1\right)_i \left(a_2\right)_i}{\left(c_1\right)_i \left(c_2\right)_i} \frac{x^i}{i!}\ {}_1F_1\left(a_1 + i; c_1 + i; y\right)\ {}_1F_1\left(a_2 + i; c_2 + i; z\right) \\ &= \sum_{i=0}^{\infty} \sum_{j=0}^{\infty} \frac{(b)_i \left(a_1\right)_{i+j} \left(a_2\right)_i}{\left(c_1\right)_{i+j} \left(c_2\right)_i} \frac{x^i y^j}{i!\ j!}\ {}_1F_1\left(a_2 + i; c_2 + i; z\right) \\ &= \sum_{i=0}^{\infty} \sum_{j=0}^{\infty} \sum_{k=0}^{\infty} \frac{(b)_i \left(a_1\right)_{i+j} \left(a_2\right)_{i+k}}{\left(c_1\right)_{i+j} \left(c_2\right)_{i+k}} \frac{x^i y^j z^k}{i!\ j!\ k!}, \end{aligned}$$

where the last two steps follow by using the series of ${}_1F_1$. Also, re-writing ${}_1F_1$ by using (A.6), an alternative series form can be derived as

$$\begin{aligned} &F_Z\left(a_1, a_2, b; c_1, c_2; x, y, z\right) \\ &= \exp(y+z) \sum_{i=0}^{\infty} \frac{(b)_i \left(a_1\right)_i \left(a_2\right)_i}{\left(c_1\right)_i \left(c_2\right)_i} \frac{x^i}{i!}\ {}_1F_1\left(c_1 - a_1; c_1 + i; -y\right)\ {}_1F_1\left(c_2 - a_2; c_2 + i; -z\right) \\ &= \exp(y+z) \sum_{i=0}^{\infty} \sum_{j=0}^{\infty} \frac{(b)_i \left(a_1\right)_i \left(a_2\right)_i \left(c_1 - a_1\right)_j}{\left(c_1\right)_{i+j} \left(c_2\right)_i} \frac{x^i (-y)^j}{i!\ j!}\ {}_1F_1\left(a_2 + i; c_2 + i; z\right) \\ &= \exp(y) \sum_{i=0}^{\infty} \sum_{j=0}^{\infty} \sum_{k=0}^{\infty} \frac{(b)_i \left(a_1\right)_i \left(a_2\right)_i \left(c_1 - a_1\right)_j \left(c_2 - a_2\right)_k}{\left(c_1\right)_{i+j} \left(c_2\right)_{i+k}} \frac{x^i (-y)^j (-z)^k}{i!\ j!\ k!}. \end{aligned}$$

Further, writing the infinite series involving the index i in terms of ${}_3F_2$, we obtain

$$F_Z\left(a_1, a_2, b; c_1, c_2; x, y, z\right) = \exp(y+z) \sum_{j=0}^{\infty} \sum_{k=0}^{\infty} \frac{\left(c_1 - a_1\right)_j \left(c_2 - a_2\right)_k}{\left(c_1\right)_j \left(c_2\right)_k} \frac{(-y)^j (-z)^k}{j!\ k!}\ {}_3F_2\left(a_1, a_2, b, ; c_1 + j, c_2 + k; x\right). \tag{A.10}$$

We can also observe that

$$F_Z\left(a_1, a_2, b; c_1, a_2; x, y, z\right) = \exp(y+z) \sum_{j=0}^{\infty} \frac{\left(c_1 - a_1\right)_j}{\left(c_1\right)_j} \frac{(-y)^j}{j!}\ {}_2F_1\left(a_1, b; c_1 + j; x\right),$$

$$\begin{aligned} F_Z\left(a_1, a_2, b; c_1, c_2; x, y, 0\right) &= \sum_{j=0}^{\infty} \frac{\left(a_1\right)_j}{\left(c_1\right)_j} \frac{y^j}{j!}\ {}_3F_2\left(a_1 + j, a_2, b; c_1 + j, c_2; x\right) \\ &= \sum_{i=0}^{\infty} \frac{(b)_i \left(a_1\right)_i \left(a_2\right)_i}{\left(c_1\right)_i \left(c_2\right)_i} \frac{x^i}{i!}\ {}_1F_1\left(a_1 + i; c_1 + i; y\right), \end{aligned}$$

$$F_Z\left(a_1, a_2, b; c_1, c_2; x, 0, 0\right) = {}_3F_2\left(a_1, a_2, b; c_1, c_2; x\right)$$

and

$$F_Z\left(a_1, a_2, b; c_1, c_2; 0, y, z\right) = {}_1F_1\left(a_1; c_1; y\right)\ {}_1F_1\left(a_2; c_2; z\right).$$

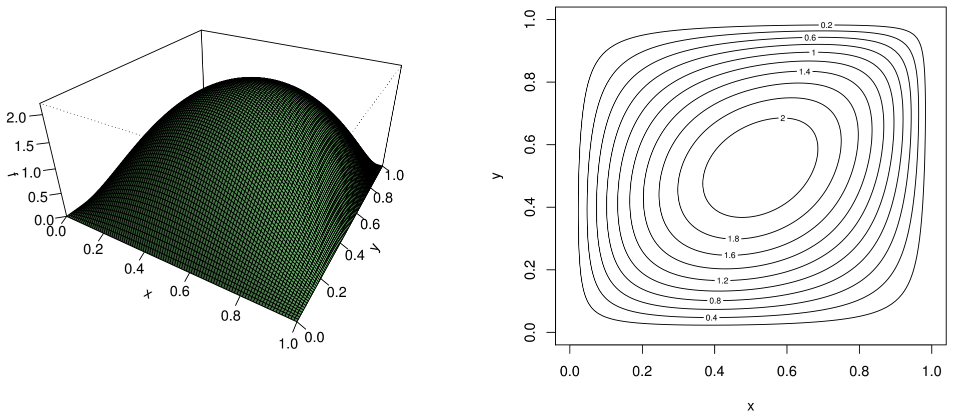

$\alpha = 2$, $\beta = 2$, $\gamma = 4$, $\sigma = 0.5$, $\lambda_1 = 1$, $\lambda_2 = 1$

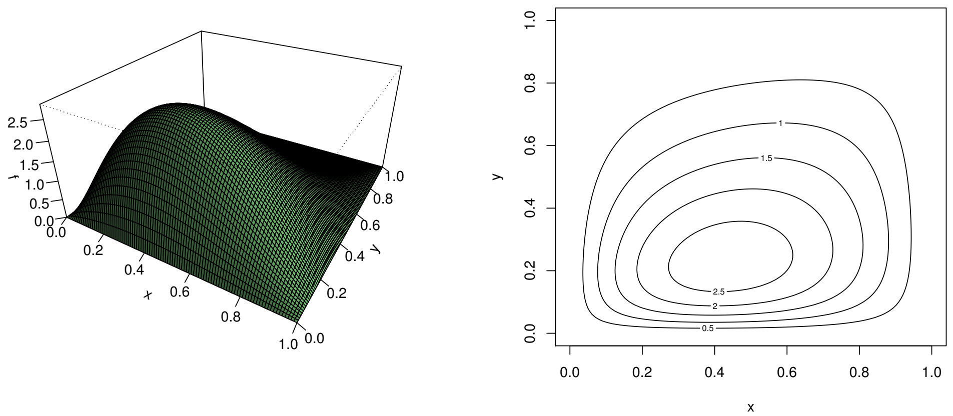

$\alpha = 2$, $\beta = 2$, $\gamma = 4$, $\sigma = 0.5$, $\lambda_1 = 1$, $\lambda_2 = 4$

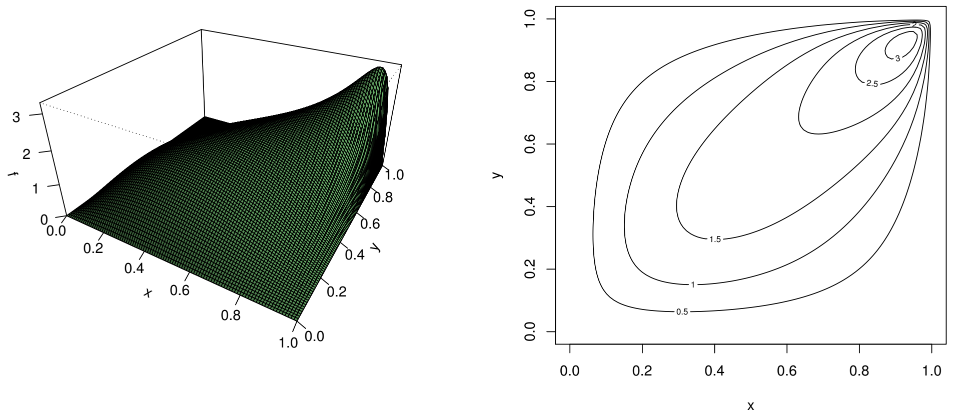

$\alpha = 2$, $\beta = 2$, $\gamma = 4$, $\sigma = 0.9$, $\lambda_1 = 2$, $\lambda_2 = 2$

Fig 1. Plots of the pdf for selected values of α, β, γ, σ, λ_1 and λ_2.

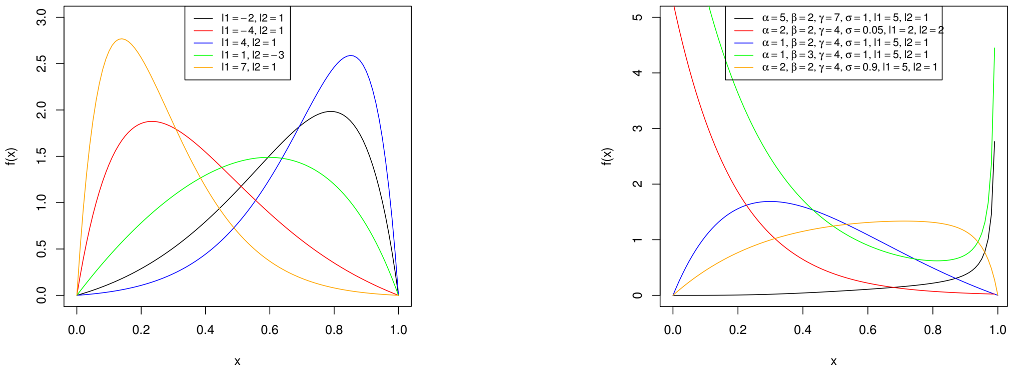

$(\alpha, \beta, \gamma, \sigma, \lambda_1, \lambda_2) = (2, 2, 4, 0.5, -2, 1),\ (2, 2, 4, 0.5, -4, 1),$
$(2, 2, 4, 0.5, 4, 1),\ (2, 2, 4, 0.5, 1, -3),\ (2, 2, 4, 0.5, 7, 1)$

$(\alpha, \beta, \gamma, \sigma, \lambda_1, \lambda_2) = (5, 2, 7, 1, 5, 1),\ (2, 2, 4, 0.05$
$(1, 2, 4, 1, 5, 1),\ (1, 3, 4, 1, 5, 1),\ (2, 2, 4, 0.9, 5, 1)$

Fig 2. Plots of the marginal pdf of X for selected values of α, β, γ, σ, λ_1, and λ_2.

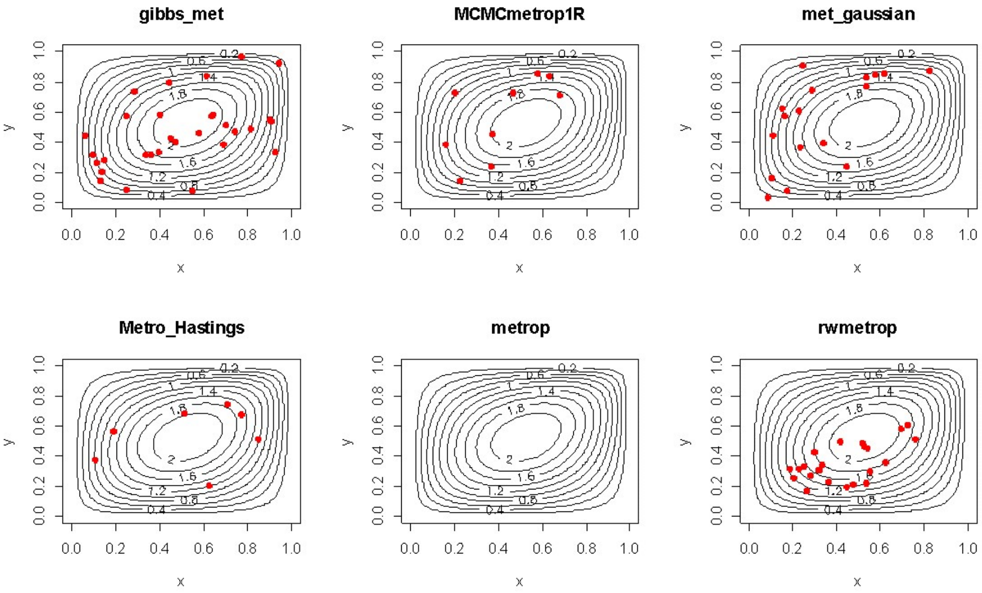

Fig 3. Simulated data and contour plots for different methods for $\alpha = 2$, $\beta = 2$, $\gamma = 4$, $\sigma = 0.5$, $\lambda_1 = 1$ and $\lambda_2 = 1$, $n = 30$.

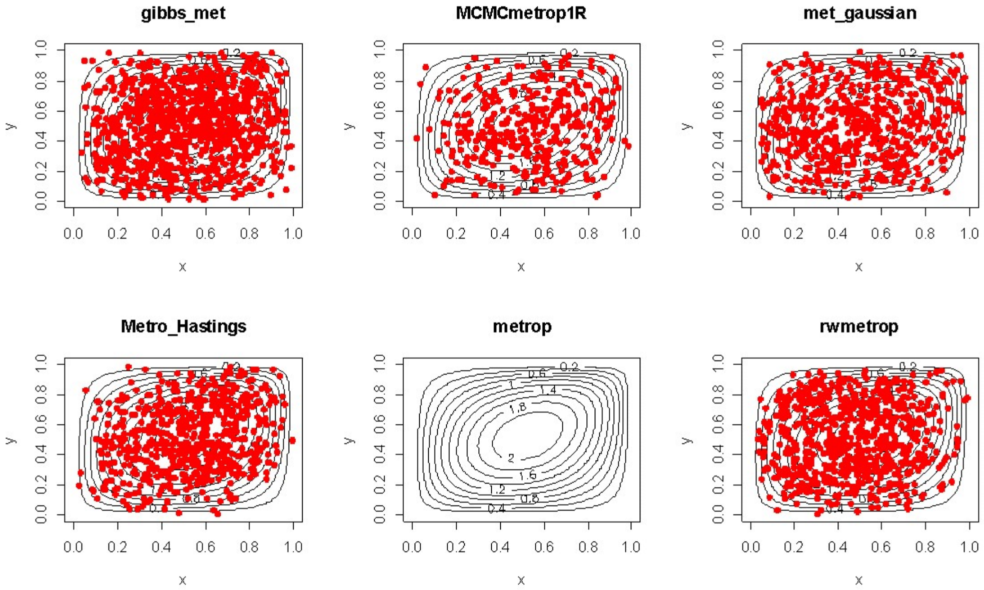

Fig 4. Simulated data and contour plots for different methods for $\alpha = 2$, $\beta = 2$, $\gamma = 4$, $\sigma = 0.5$, $\lambda_1 = 1$ and $\lambda_2 = 1$, $n = 1000$.

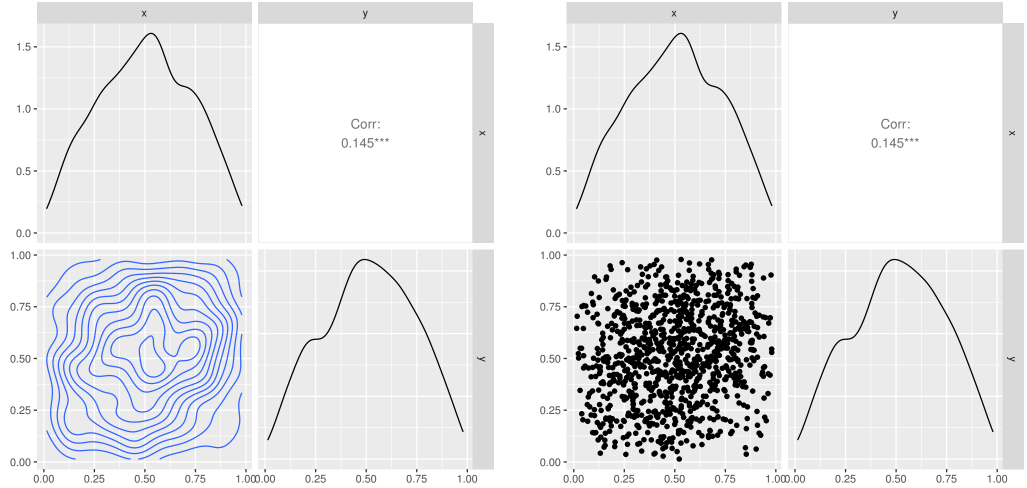

Fig 5. Pairs style of Gibbs sampling method for $\alpha = 2$, $\beta = 2$, $\gamma = 4$, $\sigma = 0.5$, $\lambda_1 = 1$ and $\lambda_2 = 1$, $n = 1000$.

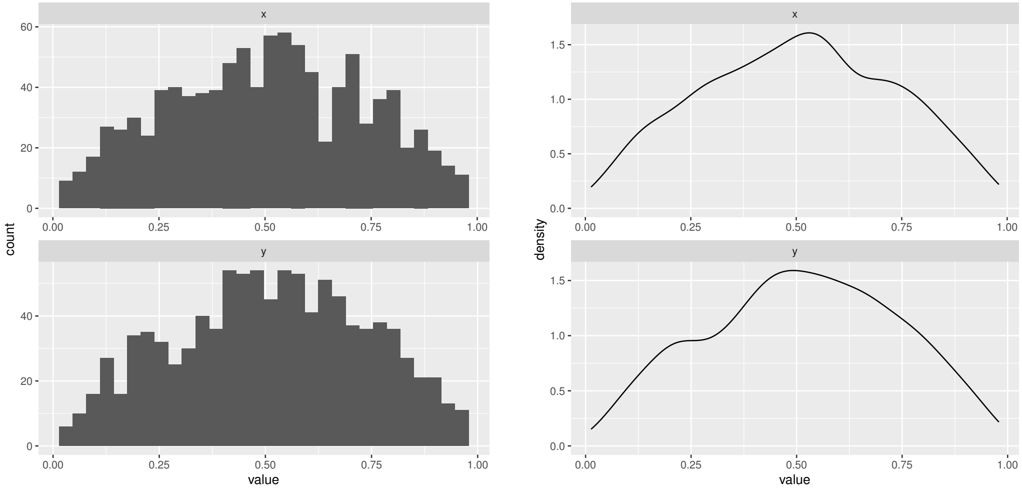

Fig 6. Histogram of simulated data and marginal pdfs for $\alpha = 2$, $\beta = 2$, $\gamma = 4$, $\sigma = 0.5$, $\lambda_1 = 1$ and $\lambda_2 = 1$, $n = 1000$.

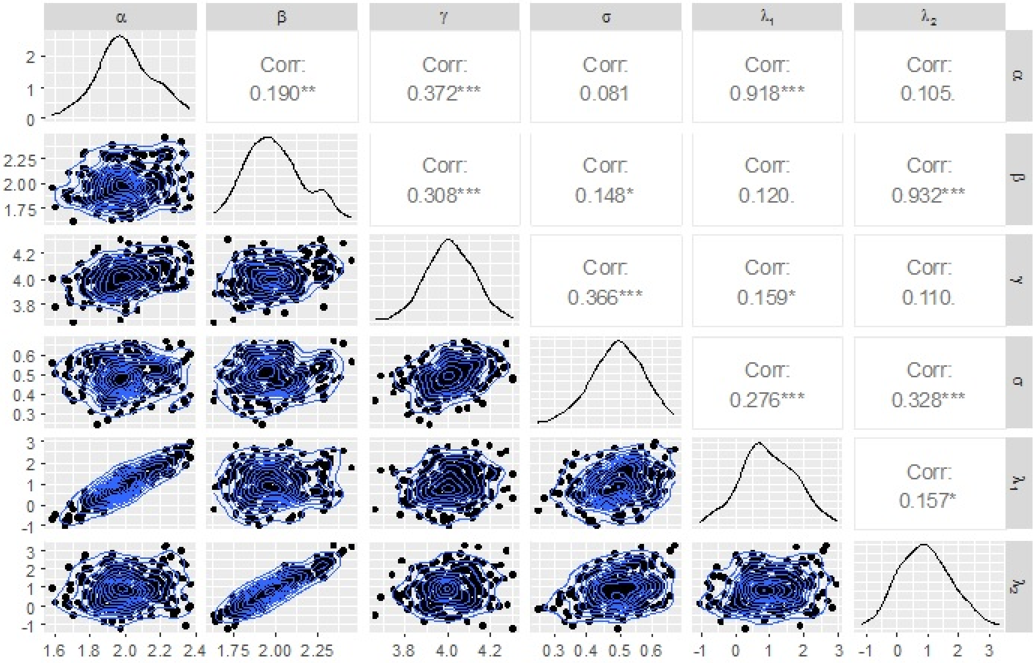

Fig 7. Correlation between X and Y for $\alpha = 2$, $\beta = 2$, $\gamma = 4$, $\sigma = 0.5$, $\lambda_1 = 1$ and $\lambda_2 = 1$, $n = 1000$.

References

1. Y. L. Luke, *The Special Functions and Their Approximations*, Volume 1, Academic Press, New York, 1969.

2. A. P. Prudnikov, Yu. A. Brychkov, and O. I. Marichev, *Integrals and Series*. Vol. 3. More Special Functions, Gordon and Breach Science, New York, NY, USA, 1990, translated from the Russian by G. G. Gould.
