# Supplementary material for: Generalized bivariate Kummer-beta distribution with marginals defined on the unit interval
Source: PLoS One. 2024 Oct 28;19(10):e0311888. doi: 10.1371/journal.pone.0311888 (PMC11516163; doi:10.1371/journal.pone.0311888)
Supplement: S2 Appendix — (PDF) [file pone.0311888.s002.pdf]

Appendix B

```
# Packages -------------------------------------------

library(mcmc) # metrop
library(MCMCpack) # MCMCmetrop1R
library(gibbs.met) # gibbs_met & met_gaussian
library(LearnBayes) # rwmetrop
library(MHadaptive) # Metro_Hastings
library(coda) # as.mcmc
library(ggmcmc) # ggs & ggs_*
library(MCMCvis) # MCMCtrace
library(microbenchmark) # microbenchmark
# library(bench) # mark
# library(ggplot2) # autoplot
library(pracma)  # integral2
# library(plyr) # raply
library(optimx) # optimx
library(dplyr)
library(GGally)

# Density and log density ------------------------------

dgbkb0<-function(x,y,alpha,beta,gama,sigma,lambda1,lambda2){
  a=alpha ; b=beta ; g=gama ; s=sigma ; l1=lambda1 ; l2=lambda2
  x^(a-1)*y^(b-1)*(1-x)^(g-a-1)*(1-y)^(g-b-1)/
    (1-s*x*y)^g*exp(-(l1*x+l2*y))
}

c_func=function(alpha,beta,gama,sigma,lambda1,lambda2){
  c0=integral2(fun=dgbkb0,xmin=0,xmax=1,ymin=0,ymax=1,
               alpha=alpha,beta=beta,gama=gama,sigma=sigma,
               lambda1=lambda1,lambda2=lambda2)$Q
  c0
}

logdgbkb0<-function(x,y,alpha,beta,gama,sigma,lambda1,lambda2){
  a=alpha ; b=beta ; g=gama ; s=sigma ; l1=lambda1 ; l2=lambda2
  (a-1)*log(x)+(b-1)*log(y)+(g-a-1)*log(1-x)+(g-b-1)*log(1-y)-
    g*log(1-s*x*y)-(l1*x+l2*y)
}

dgbkb<-function(xy,alpha,beta,gama,sigma,lambda1,lambda2,c0){
  a=alpha ; b=beta ; g=gama ; s=sigma ; l1=lambda1 ; l2=lambda2
  x=xy[1]; y=xy[2]
  f<-ifelse(
    all(xy > 0 & xy < 1),
    1/c0*dgbkb0(x,y,alpha,beta,gama,sigma,lambda1,lambda2),
    0
  )
  return(f)
}
```

```
dgbkb2<-function(x,y,alpha,beta,gama,sigma,lambda1,lambda2,c0) {
  a=alpha ; b=beta ; g=gama ; s=sigma ; l1=lambda1 ; l2=lambda2
  f=1/c0*dgbkb0(x,y,alpha,beta,gama,sigma,lambda1,lambda2)
  return(f)
}

logdgbkb<-function(xy,alpha,beta,gama,sigma,lambda1,lambda2,c0) {
  x=xy[1]; y=xy[2]
  logl<-ifelse(
    all(xy > 0 & xy < 1),
    -log(c0)+logdgbkb0(x,y,alpha,beta,gama,sigma,lambda1,lambda2),
    -Inf
  )
  return(logl)
}

logdgbkb2<-function(x,y,alpha,beta,gama,sigma,lambda1,lambda2,c0) {
  logl=-log(c0)+logdgbkb0(x,y,alpha,beta,gama,sigma,lambda1,lambda2)
  return(logl)
}

mloglgbkb=function(param,x,y,c0=NULL){
  alpha=param[1]
  beta=param[2]
  gama=param[3]
  sigma=param[4]
  lambda1=param[5]
  lambda2=param[6]
  if(gama<=alpha | gama<=beta) return(Inf)
  if(is.null(c0)){
    c0=c_func(alpha,beta,gama,sigma,lambda1,lambda2)
  }
  m=-sum(logdgbkb2(x,y,alpha,beta,gama,sigma,lambda1,lambda2,c0))
  # print(param)
  return(m)
}

mloglgbkb2=function(alpha,beta,gama,sigma,lambda1,lambda2,x,y,c0=NULL){
  if(gama<=alpha | gama<=beta) return(Inf)
  if(is.null(c0)){
    c0=c_func(alpha,beta,gama,sigma,lambda1,lambda2)
  }
  m=-sum(logdgbkb2(x,y,alpha,beta,gama,sigma,lambda1,lambda2,c0))
  return(m)
}

fitgbkb=function(param,x,y){
  fit=optim(par=p0,fn=mloglgbkb,x=x,y=y,hessian=T,
            lower=c(0,0,0,0,-Inf,-Inf),upper=c(Inf,Inf,Inf,1,Inf,Inf),
            control=list(maxit=1000),
            method="L-BFGS-B")
  phat=fit$par
```

```
  h=fit$hessian
  v=abs(solve(h))
  bias=phat-param
  names(bias)=paste("bias",names(bias),sep="_")
  stdev=sqrt(diag(v))
  names(stdev)=paste("sd",names(stdev),sep="_")

  out=c(phat,bias,stdev)

  # MSE=diag(v)+bias^2
  # names(MSE)=paste("MSE",names(MSE),sep="_")
  # out=c(phat,bias,stdev,MSE)

  return(out)
}

sim_mle_gbkb=function(n,Bp,xy0,alpha,beta,gama,sigma,lambda1,lambda2,c0){
  p0=c(alpha=alpha,beta=beta,gama=gama,sigma=sigma,
  lambda1=lambda1,lambda2=lambda2)
  xy=rgbkb(n=n,Bp=Bp,xy0=xy0,alpha=alpha,beta=beta,
  gama=gama,sigma=sigma,
  lambda1=lambda1,lambda2=lambda2,c0=c0,method="gibbs_met")
  x=xy[,1]
  y=xy[,2]
  fit=fitgbkb(p0,x,y)
  return(fit)
}

# Random Generator ---------------------------------------------------------

rgbkb1_rwmetrop<-function(n,Bp,xy0,alpha,beta,gama,sigma,
lambda1,lambda2,c0) {
  proposal<-list(var=diag(2),scale=1)
  xy<-rwmetrop(
    logpost=logdgbkb,proposal=proposal,start=xy0,m=n+Bp,
    alpha=alpha,beta=beta,gama=gama,sigma=sigma,
    lambda1=lambda1,lambda2=lambda2,c0=c0
  )
  xy<-xy$par[-(1:Bp),]
  if (is.null(names(xy0))) {
    colnames(xy)<-c("x","y")
  } else {
    colnames(xy)<-names(xy0)
  }

  return(xy)
}

rgbkb_rwmetrop<-function(n,Bp,xy0,alpha,beta,gama,sigma,
lambda1,lambda2,c0) {
  proposal<-list(var=diag(2),scale=0.5)
  xy<-rwmetrop(
```

```
    logpost=logdgbkb,proposal=proposal,start=xy0,m=n+Bp,
    alpha=alpha,beta=beta,gama=gama,sigma=sigma,
    lambda1=lambda1,lambda2=lambda2,c0=c0
  )
  v<-var(xy$par)
  proposal<-list(var=v,scale=0.5)
  xy<-rwmetrop(
    logpost=logdgbkb,proposal=proposal,start=xy0,m=n+Bp,
    alpha=alpha,beta=beta,gama=gama,sigma=sigma,lambda1=lambda1,
    lambda2=lambda2,c0=c0
  )
  xy<-xy$par[-(1:Bp),]
  if (is.null(names(xy0))) {
    colnames(xy)<-c("x","y")
  } else {
    colnames(xy)<-names(xy0)
  }
  return(xy)
}

rgbkb1_Metro_Hastings<-function(n,Bp,xy0,alpha,beta,
gama,sigma,lambda1,lambda2,c0) {
  xy<-Metro_Hastings(
    li_func=logdgbkb,pars=xy0,par_names=c("x","y"),quiet=T,
    iterations=n+Bp,burn_in=Bp,alpha=alpha,beta=beta,
    gama=gama,sigma=sigma,
    lambda1=lambda1,lambda2=lambda2,c0=c0
  )
  xy<-xy$trace[1:n,]
  if (is.null(names(xy0))) {
    colnames(xy)<-c("x","y")
  } else {
    colnames(xy)<-names(xy0)
  }
  return(xy)
}

rgbkb_Metro_Hastings<-function(n,Bp,xy0,alpha,beta,gama,
sigma,lambda1,lambda2,c0) {
  xy<-Metro_Hastings(
    li_func=logdgbkb,pars=xy0,par_names=c("x","y"),quiet=T,
    iterations=n+Bp,burn_in=Bp,alpha=alpha,beta=beta,
    gama=gama,sigma=sigma,
    lambda1=lambda1,lambda2=lambda2,c0=c0
  )
  xy<-Metro_Hastings(
    li_func=logdgbkb,pars=xy0,par_names=c("x","y"),quiet=T,
    iterations=n+Bp,burn_in=Bp,alpha=alpha,beta=beta,
    gama=gama,sigma=sigma,
    lambda1=lambda1,lambda2=lambda2,c0=c0
  )
  xy<-xy$trace[1:n,]
```

```
  if (is.null(names(xy0))) {
    colnames(xy)<-c("x","y")
  } else {
    colnames(xy)<-names(xy0)
  }
  return(xy)
}


rgbkb_MCMCmetrop1R<-function(n,Bp,xy0,alpha,beta,gama,
sigma,lambda1,lambda2,c0) {
  xy_mcmc<-MCMCmetrop1R(
    fun=dgbkb,theta.init=xy0,burnin=Bp,mcmc=n,logfun=F,
    alpha=alpha,beta=beta,gama=gama,sigma=sigma,lambda1=lambda1,
    lambda2=lambda2,c0=c0,seed=rpois(n=1,lambda=12345)
  )
  xy<-xy_mcmc
  if (is.null(names(xy0))) {
    colnames(xy)<-c("x","y")
  } else {
    colnames(xy)<-names(xy0)
  }
  return(xy)
}

rgbkb_gibbs_met<-function(n,Bp,xy0,alpha,beta,gama,
sigma,lambda1,lambda2,c0) {
  xy_mcmc<-gibbs_met(
    log_f=logdgbkb,no_var=2,ini_value=xy0,iters=n +
      Bp,iters_met=10,stepsizes_met=c(0.5,0.5),
    alpha=alpha,beta=beta,gama=gama,sigma=sigma,
    lambda1=lambda1,lambda2=lambda2,c0=c0
  )
  xy<-xy_mcmc[-(1:(Bp+1)),]
  if (is.null(names(xy0))) {
    colnames(xy)<-c("x","y")
  } else {
    colnames(xy)<-names(xy0)
  }
  return(xy)
}

rgbkb_met_gaussian<-function(n,Bp,xy0,alpha,beta,gama,
sigma,lambda1,lambda2,c0) {
  xy_mcmc<-met_gaussian(
    log_f=logdgbkb,no_var=2,ini_value=xy0,iters=n +
      Bp,iters_per.iter=10,stepsizes_met=c(1,1),
    alpha=alpha,beta=beta,gama=gama,sigma=sigma,
    lambda1=lambda1,lambda2=lambda2,c0=c0
  )
  xy<-xy_mcmc[-(1:(Bp+1)),]
  if (is.null(names(xy0))) {
```

```
    colnames(xy)<-c("x","y")
  } else {
    colnames(xy)<-names(xy0)
  }
  return(xy)
}

rgbkb1_metrop<-function(n,Bp,xy0,alpha,beta,gama,sigma,
lambda1,lambda2,c0) {
  xy_mcmc<-metrop(
    obj=dgbkb,initial=xy0,nbatch=n+Bp,blen=1,scale=0.5,
    alpha=alpha,beta=beta,gama=gama,sigma=sigma,
    lambda1=lambda1,lambda2=lambda2,c0=c0
  )
  xy<-xy_mcmc$batch[-(1:Bp),]
  if (is.null(names(xy0))) {
    colnames(xy)<-c("x","y")
  } else {
    colnames(xy)<-names(xy0)
  }
  return(xy)
}

rgbkb_metrop<-function(n,Bp,xy0,alpha,beta,gama,sigma,
lambda1,lambda2,c0) {
  xy_mcmc<-metrop(
    obj=dgbkb,initial=xy0,nbatch=n+Bp,blen=1,scale=0.5,
    alpha=alpha,beta=beta,gama=gama,sigma=sigma,
    lambda1=lambda1,
    lambda2=lambda2,c0=c0
  )
  xy_mcmc<-metrop(
    obj=xy_mcmc,initial=xy0,nbatch=n+Bp,blen=1,scale=0.5,
    alpha=alpha,beta=beta,gama=gama,sigma=sigma,
    lambda1=lambda1,
    lambda2=lambda2,c0=c0
  )
  xy<-xy_mcmc$batch[-(1:Bp),]
  if (is.null(names(xy0))) {
    colnames(xy)<-c("x","y")
  } else {
    colnames(xy)<-names(xy0)
  }
  return(xy)
}

rgbkb<-function(n,Bp,xy0,alpha,beta,gama,sigma,lambda1,
lambda2,c0,method="rwmetrop") {
  methods<-substr(apropos("rgbkb_"),start=7,stop=20)
  if (!(method %in% methods)) {
    stop("argument 'method' must be one of defined methods.
    \n See:  substr(apropos('rgbkb_'),start=6,stop=20)")
```

```
  }
  rfunc<-match.fun(paste("rgbkb",method,sep="_"))
  xy<-rfunc(n,Bp,xy0,alpha,beta,gama,sigma,lambda1,lambda2,c0)
  if (is.null(names(xy0))) {
    colnames(xy)<-c("x","y")
  } else {
    colnames(xy)<-names(xy0)
  }
  return(xy)
}


# Density 2D -------------------------------------------------

dgbkb0<-function(x,y,alpha,beta,gama,sigma,lambda1,lambda2){
  a=alpha ; b=beta ; g=gama ; s=sigma ; l1=lambda1 ; l2=lambda2
  x^(a-1)*y^(b-1)*(1-x)^(g-a-1)*(1-y)^(g-b-1)/
    (1-s*x*y)^g*exp(-(l1*x+l2*y))
}

c_func=function(alpha,beta,gama,sigma,lambda1,lambda2){
  c0=integral2(fun=dgbkb0,xmin=0,xmax=1,ymin=0,ymax=1,
               alpha=alpha,beta=beta,gama=gama,sigma=sigma,
               lambda1=lambda1,lambda2=lambda2)$Q
  c0
}

dgbkb<-function(xy,alpha,beta,gama,sigma,lambda1,lambda2,c0){
  a=alpha ; b=beta ; g=gama ; s=sigma ; l1=lambda1 ;
  l2=lambda2
  x=xy[1]; y=xy[2]
  f<-ifelse(
    all(xy > 0 & xy < 1),
    1/c0*dgbkb0(x,y,alpha,beta,gama,sigma,lambda1,lambda2),
    0
  )
  return(f)
}

dgbkb2<-function(x,y,alpha,beta,gama,sigma,lambda1,lambda2,c0) {
  a=alpha ; b=beta ; g=gama ; s=sigma ; l1=lambda1 ;
  l2=lambda2
  f=1/c0*dgbkb0(x,y,alpha,beta,gama,sigma,
  lambda1,lambda2)
  return(f)
}

# Marginal --------------------------------------

dbaseX <- function(x,alpha,beta,gama,sigma,lambda1,lambda2,c0){
  integrate(
    f = dgbkb2,
```

```
    lower = 0,
    upper = 1,
    x=x, alpha=alpha,beta=beta,gama=gama,
    sigma=sigma,lambda1=lambda1,lambda2=lambda2,
    c0=c0
  )$value
}

dgkbX <- Vectorize(dbaseX,"x")

dbaseY <- function(y,alpha,beta,gama,sigma,lambda1,
lambda2,c0){
  integrate(
    f = dgbkb2,
    lower = 0,
    upper = 1,
    y=y, alpha=alpha,beta=beta,gama=gama,
    sigma=sigma,lambda1=lambda1,lambda2=lambda2,
    c0=c0
  )$value
}

dgkbY <- Vectorize(dbaseY,"y")

# Parent Density Single ---------------------------------------

B=100

# choose one set:
alpha=2 ; beta=2 ; gama=4 ; sigma=0.5 ; lambda1=1 ;
lambda2=1
# alpha=2 ; beta=2 ; gama=6 ; sigma=0.5 ;
# lambda1=2 ; lambda2=2
# alpha=2 ; beta=2 ; gama=4 ; sigma=0.5 ;
# lambda1=1.5 ; lambda2=1.5

p0=c(alpha=alpha,beta=beta,gama=gama,sigma=sigma,
lambda1=lambda1,lambda2=lambda2)
a=alpha ; b=beta ; g=gama ; s=sigma ; l1=lambda1 ;
l2=lambda2
c0=c_func(alpha,beta,gama,sigma,lambda1,lambda2)
c0

# Then:
x=seq(0,1,length=B)
y=seq(0,1,length=B)
f=outer(x,y,dgbkb2,alpha=alpha,beta=beta,gama=gama,
sigma=sigma,lambda1=lambda1,lambda2=lambda2,c0=c0)
par(mfrow=c(1,1))
persp(x,y,f,
      theta=30,phi=30,expand=0.5,col="lightgreen",ltheta=120,
      shade=0.75,ticktype="detailed"
```

```
)
contour(x,y,f,xlab="x",ylab="y")
# legend("topright",ex,
#        col=c("black","red","blue","green","orange"),cex=0.8,
#        text.width=strwidth("1,000,000,00000")
# )
par(mfrow=c(1,1))

p0
integral2(fun=dgbkb2,xmin=0,xmax=1,ymin=0,ymax=1,alpha=alpha,
beta=beta,gama=gama,sigma=sigma,lambda1=lambda1,
lambda2=lambda2,c0=c0)
cubature::hcubature(f=dgbkb,lowerLimit=rep(0,2),
upperLimit=rep(1,2),
alpha=alpha,beta=beta,gama=gama,sigma=sigma,lambda1=lambda1,
lambda2=lambda2,c0=c0,tol=1e-7)

# Parent Density Multiple ---------------------

B=100

parlist=list(
  list(alpha=2 , beta=2 , gama=4 , sigma=0.5 ,
  lambda1=1 , lambda2=1),
  list(alpha=2 , beta=2 , gama=6 , sigma=0.5 ,
  lambda1=2 , lambda2=2),
  list(alpha=2 , beta=2 , gama=4 , sigma=0.5 ,
  lambda1=1.5 , lambda2=1.5)
)

# choose one panel:
par(mfrow=c(3,2))
par(mfrow=c(1,2))

# Then:
for (i in 1:3) {
  param=parlist[[i]]
  alpha=param$alpha
  beta=param$beta
  gama=param$gama
  sigma=param$sigma
  lambda1=param$lambda1
  lambda2=param$lambda2
  c0=c_func(alpha,beta,gama,sigma,lambda1,lambda2)
  x=seq(0,1,length=B)
  y=seq(0,1,length=B)
  f=outer(x,y,dgbkb2,alpha=alpha,beta=beta,gama=gama,
  sigma=sigma,lambda1=lambda1,lambda2=lambda2,c0=c0)
  persp(x,y,f,
        theta=30,phi=30,expand=0.5,col="lightgreen",
        ltheta=120,
        shade=0.75,ticktype="detailed"
```

```
  )
  contour(x,y,f,xlab="x",ylab="y")
}
par(mfrow=c(1,1))

# Comparing Simulation Functions ----------------------

# All Functions:
apropos("rgbkb_")
methods=substr(apropos("rgbkb_"),start=7,stop=20)
methods

# number of simulation
n=1000
# number of burning
Bp=100
# initial value:
xy0=c(x=0.5,y=0.5)

# choose one set:
alpha=2 ; beta=2 ; gama=4 ; sigma=0.5 ;
lambda1=1 ; lambda2=1
# alpha=2 ; beta=2 ; gama=6 ; sigma=0.5 ;
# lambda1=2 ; lambda2=2
# alpha=2 ; beta=2 ; gama=4 ; sigma=0.5 ;
# lambda1=1.5 ; lambda2=1.5

p0=c(alpha=alpha,beta=beta,gama=gama,sigma=sigma,
lambda1=lambda1,lambda2=lambda2)
a=alpha ; b=beta ; g=gama ; s=sigma ;
l1=lambda1 ; l2=lambda2

c0=c_func(alpha,beta,gama,sigma,lambda1,lambda2)

B=100
x=seq(0,1,length=B)
y=seq(0,1,length=B)
f=outer(x,y,dgbkb2,alpha=alpha,beta=beta,gama=gama,
sigma=sigma,lambda1=lambda1,lambda2=lambda2,c0=c0)
par(mfrow=c(1,2))
persp(x,y,f,
      theta=30,phi=30,expand=0.5,col="lightgreen",
      ltheta=120,
      shade=0.75,ticktype="detailed"
)
contour(x,y,f,xlab="x",ylab="y")
par(mfrow=c(1,1))

# All Methods:
i=0

i=i+1; i # till 6
```

```
method=methods[i]; method
xy=rgbkb(n=n,Bp=Bp,xy0=xy0,alpha=alpha,beta=beta,gama=gama,
sigma=sigma,lambda1=lambda1,lambda2=lambda2,c0=c0,
method=method)
dim(xy) # n*2
par(mfrow=c(1,1))
contour(x,y,f,xlab="x",ylab="y")
points(xy,col="red",pch=19)
MCMCtrace(xy,pdf=F)
S=ggs(as.mcmc(xy))
ggs_histogram(S)
ggs_density(S)
ggs_traceplot(S)
ggs_compare_partial(S)
ggs_autocorrelation(S)
ggs_crosscorrelation(S)
ggs_pairs(S)
ggs_pairs(S,lower=list(continuous="density"))
ggs_pairs(S,upper=list(continuous="density"))
i # till 6
method

# Comparing Time:
apropos("rgbkb_")
apropos("rgbkb1_")
# number of simulation
n=100
# number of burning
Bp=100

# choose one set:
alpha=2 ; beta=2 ; gama=4 ; sigma=0.5 ;
lambda1=1 ; lambda2=1
# alpha=2 ; beta=2 ; gama=6 ; sigma=0.5 ;
# lambda1=2 ; lambda2=2
# alpha=2 ; beta=2 ; gama=4 ; sigma=0.5 ;
# lambda1=1.5 ; lambda2=1.5

p0=c(alpha=alpha,beta=beta,gama=gama,sigma=sigma,
lambda1=lambda1,lambda2=lambda2,c0=c0)
a=alpha ; b=beta ; g=gama ; s=sigma ; l1=lambda1 ;
l2=lambda2
c0=c_func(alpha,beta,gama,sigma,lambda1,lambda2)

tm1=microbenchmark(
  xy_MCMCmetrop1R=rgbkb_MCMCmetrop1R(n=n,Bp=Bp,xy0=xy0,
  alpha=alpha,beta=beta,gama=gama,sigma=sigma,
  lambda1=lambda1,lambda2=lambda2,c0=c0),
  xy_rwmetrop1=rgbkb1_rwmetrop(n=n,Bp=Bp,xy0=xy0,
  alpha=alpha,beta=beta,gama=gama,sigma=sigma,
  lambda1=lambda1,lambda2=lambda2,c0=c0),
  xy_rwmetrop=rgbkb_rwmetrop(n=n,Bp=Bp,xy0=xy0,
```

```
  alpha=alpha,beta=beta,gama=gama,sigma=sigma,
  lambda1=lambda1,lambda2=lambda2,c0=c0),
  xy_Metro_Hastings1=rgbkb1_Metro_Hastings(n=n,
  Bp=Bp,xy0=xy0,alpha=alpha,beta=beta,gama=gama,
  sigma=sigma,lambda1=lambda1,lambda2=lambda2,c0=c0),
  xy_met_gaussian=rgbkb_met_gaussian(n=n,Bp=Bp,
  xy0=xy0,alpha=alpha,beta=beta,gama=gama,
  sigma=sigma,lambda1=lambda1,lambda2=lambda2,c0=c0),
  xy_Metro_Hastings=rgbkb_Metro_Hastings(n=n,
  Bp=Bp,xy0=xy0,alpha=alpha,beta=beta,gama=gama,
  sigma=sigma,lambda1=lambda1,lambda2=lambda2,c0=c0),
  xy_gibbs_met=rgbkb_gibbs_met(n=n,Bp=Bp,xy0=xy0,
  alpha=alpha,beta=beta,gama=gama,sigma=sigma,
  lambda1=lambda1,lambda2=lambda2,c0=c0),
  xy_metrop1=rgbkb1_metrop(n=n,Bp=Bp,xy0=xy0,
  alpha=alpha,beta=beta,gama=gama,sigma=sigma,
  lambda1=lambda1,lambda2=lambda2,c0=c0),
  xy_metrop=rgbkb_metrop(n=n,Bp=Bp,xy0=xy0,
  alpha=alpha,beta=beta,gama=gama,sigma=sigma,
  lambda1=lambda1,lambda2=lambda2,c0=c0)
)
tm1
autoplot(tm1)
boxplot(tm1)

tm2=microbenchmark(
  xy_MCMCmetrop1R=rgbkb_MCMCmetrop1R(n=n,Bp=Bp,
  xy0=xy0,alpha=alpha,beta=beta,gama=gama,
  sigma=sigma,lambda1=lambda1,lambda2=lambda2,c0=c0),
  xy_rwmetrop=rgbkb_rwmetrop(n=n,Bp=Bp,xy0=xy0,
  alpha=alpha,beta=beta,gama=gama,sigma=sigma,
  lambda1=lambda1,lambda2=lambda2,c0=c0),
  xy_met_gaussian=rgbkb_met_gaussian(n=n,Bp=Bp,
  xy0=xy0,alpha=alpha,beta=beta,gama=gama,
  sigma=sigma,lambda1=lambda1,lambda2=lambda2,c0=c0),
  xy_Metro_Hastings=rgbkb_Metro_Hastings(n=n,
  Bp=Bp,xy0=xy0,alpha=alpha,beta=beta,gama=gama,
  sigma=sigma,lambda1=lambda1,lambda2=lambda2,c0=c0),
  xy_gibbs_met=rgbkb_gibbs_met(n=n,Bp=Bp,
  xy0=xy0,alpha=alpha,beta=beta,gama=gama,
  sigma=sigma,lambda1=lambda1,lambda2=lambda2,c0=c0),
  xy_metrop=rgbkb_metrop(n=n,Bp=Bp,
  xy0=xy0,alpha=alpha,beta=beta,gama=gama,
  sigma=sigma,lambda1=lambda1,lambda2=lambda2,c0=c0)
)
tm2
autoplot(tm2)
boxplot(tm2)

tm3=microbenchmark(
  xy_MCMCmetrop1R=rgbkb_MCMCmetrop1R(n=n,
  Bp=Bp,xy0=xy0,alpha=alpha,beta=beta,
```

```
  gama=gama,sigma=sigma,lambda1=lambda1,
  lambda2=lambda2,c0=c0),
  xy_met_gaussian=rgbkb_met_gaussian(n=n,
  Bp=Bp,xy0=xy0,alpha=alpha,beta=beta,
  gama=gama,sigma=sigma,lambda1=lambda1,
  lambda2=lambda2,c0=c0),
  xy_Metro_Hastings=rgbkb_Metro_Hastings(n=n
  ,Bp=Bp,xy0=xy0,alpha=alpha,beta=beta,
  gama=gama,sigma=sigma,lambda1=lambda1,
  lambda2=lambda2,c0=c0),
  xy_gibbs_met=rgbkb_gibbs_met(n=n,
  Bp=Bp,xy0=xy0,alpha=alpha,beta=beta,
  gama=gama,sigma=sigma,lambda1=lambda1,
  lambda2=lambda2,c0=c0),
  xy_metrop=rgbkb_metrop(n=n,Bp=Bp,
  xy0=xy0,alpha=alpha,beta=beta,
  gama=gama,sigma=sigma,lambda1=lambda1,
  lambda2=lambda2,c0=c0)
)
tm3
autoplot(tm3)
boxplot(tm3)

# Comparing with Parent

# number of simulation
n=30
n=1000
# number of burning
Bp=100

# choose one set:
alpha=2 ; beta=2 ; gama=4 ; sigma=0.5 ;
lambda1=1 ; lambda2=1
# alpha=2 ; beta=2 ; gama=6 ; sigma=0.5 ;
# lambda1=2 ; lambda2=2
# alpha=2 ; beta=2 ; gama=4 ; sigma=0.5 ;
# lambda1=1.5 ; lambda2=1.5

p0=c(alpha=alpha,beta=beta,gama=gama,
sigma=sigma,lambda1=lambda1,lambda2=lambda2)
a=alpha ; b=beta ; g=gama ; s=sigma ;
l1=lambda1 ; l2=lambda2
c0=c_func(alpha,beta,gama,sigma,lambda1,
lambda2)

B=100
x=seq(0,1,length=B)
y=seq(0,1,length=B)
f=outer(x,y,dgbkb2,alpha=alpha,beta=beta,
gama=gama,sigma=sigma,lambda1=lambda1,
lambda2=lambda2,c0=c0)
```

```
par(mfrow=c(2,3))
for (i in 1:6) {
  set.seed(123)
  method=methods[i]
  xy=rgbkb(n=n,Bp=Bp,xy0=xy0,alpha=alpha,
  beta=beta,gama=gama,sigma=sigma,lambda1=lambda1,
  lambda2=lambda2,c0=c0,method=method)
  contour(x,y,f,xlab="x",ylab="y",
  main=method,nlevels=10)
  points(xy,col="red",pch=19)
}
par(mfrow=c(1,1))

par(mfrow=c(2,3))
for (i in 1:6) {
  set.seed(123)
  method=methods[i]
  xy=rgbkb(n=n,Bp=Bp,xy0=xy0,alpha=alpha,
  beta=beta,gama=gama,sigma=sigma,
  lambda1=lambda1,lambda2=lambda2,c0=c0,method=method)
  plot(as.matrix(xy),main=method)
}
par(mfrow=c(1,1))

# MLE Example ----------------------------

# number of simulation
n=1000
n=30
# number of burning
Bp=100
# initial value:
xy0=c(x=0.5,y=0.5)

# choose one set:
alpha=2 ; beta=2 ; gama=4 ; sigma=0.5 ;
lambda1=1 ; lambda2=1
# alpha=2 ; beta=2 ; gama=6 ;
# sigma=0.5 ; lambda1=2 ; lambda2=2
# alpha=2 ; beta=2 ; gama=4 ;
# sigma=0.5 ; lambda1=1.5 ; lambda2=1.5

p0=c(alpha=alpha,beta=beta,gama=gama,
sigma=sigma,lambda1=lambda1,lambda2=lambda2)
a=alpha ; b=beta ; g=gama ; s=sigma ;
l1=lambda1 ; l2=lambda2

c0=c_func(alpha,beta,gama,sigma,lambda1,lambda2)

xy=rgbkb(n=n,Bp=Bp,xy0=xy0,alpha=alpha,
beta=beta,gama=gama,sigma=sigma,lambda1=lambda1,
```

```
lambda2=lambda2,c0=c0,method="gibbs_met")

dim(xy) # n*2
cor(xy)

B=100
x=seq(0,1,length=B)
y=seq(0,1,length=B)
f=outer(x,y,dgbkb2,alpha=alpha,beta=beta,
gama=gama,sigma=sigma,lambda1=lambda1,
lambda2=lambda2,c0=c0)
par(mfrow=c(1,1))
contour(x,y,f,xlab="x",ylab="y")
points(xy,col="red",pch=19)

x=xy[,1]
y=xy[,2]

mloglgbkb(p0,x,y)
mloglgbkb(p0,x,y,c0)

fit0=optimx(par=p0,fn=mloglgbkb,
x=x,y=y,hessian=T,
control=list(all.methods=TRUE,
          save.failures=TRUE, trace=0))
fit0
fit0 %>%
  filter(convcode==0)

fit=optim(par=p0,fn=mloglgbkb,x=x,y=y,
hessian=T,
          lower=c(0,0,0,0,-Inf,-Inf),
          upper=c(Inf,Inf,Inf,1,Inf,Inf),
          control=list(maxit=1000),
          method="L-BFGS-B")
p0
fit

h=fit$hessian
v=solve(h)
v

fit$value

p0
fit$par
phat=fitgbkb(p0,x,y); phat

# MLE Main -----------------------

# number of simulation
n=30
```

```
n=1000
# number of burning
Bp=100
# initial value:
xy0=c(x=0.5,y=0.5)

# choose one set:
alpha=2 ; beta=2 ; gama=4 ; sigma=0.5 ;
lambda1=1 ; lambda2=1
# alpha=2 ; beta=2 ; gama=6 ;
# sigma=0.5 ; lambda1=2 ; lambda2=2
# alpha=2 ; beta=2 ; gama=4 ;
# sigma=0.5 ; lambda1=1.5 ; lambda2=1.5

p0=c(alpha=alpha,beta=beta,gama=gama,
sigma=sigma,lambda1=lambda1,lambda2=lambda2)
a=alpha ; b=beta ; g=gama ; s=sigma ;
l1=lambda1 ; l2=lambda2

c0=c_func(alpha,beta,gama,sigma,lambda1,lambda2)

xy=rgbkb(n=n,Bp=Bp,xy0=xy0,alpha=alpha,beta=beta,
gama=gama,sigma=sigma,lambda1=lambda1,
lambda2=lambda2,c0=c0,method="gibbs_met")
x=xy[,1]
y=xy[,2]

fit1=fitgbkb(param=p0,x,y)
fit1

sim1=sim_mle_gbkb(n,Bp,xy0,alpha,beta,
gama,sigma,lambda1,lambda2,c0)
sim1

npar=length(p0); npar

repl=10
set.seed(123)
sim2=plyr::raply(repl,sim_mle_gbkb(n,Bp,xy0,
alpha,beta,gama,sigma,lambda1,lambda2,c0),
.progress="text")
sim2
sim2est=sim2[,1:npar]
sim2bias=sim2[,(npar+1):(2*npar)]
sim2sd=sim2[,(2*npar+1):(3*npar)]

repl
sim2est
sim2bias
sim2sd
colMeans(sim2)
```

```
apply(sim2est,2,sd)
sim2d=sweep(sim2est,2,p0,"-")
colMeans(sim2d)
colMeans(simdbias)
mse=colMeans(sim2d^2); mse

# repl=250
repl=100
set.seed(123)
sim2=plyr::raply(repl,sim_mle_gbkb(n,Bp,xy0,
alpha,beta,gama,sigma,lambda1,lambda2,c0),
.progress="text")
sim2est=sim2[,1:npar]
sim2bias=sim2[,(npar+1):(2*npar)]
sim2sd=sim2[,(2*npar+1):(3*npar)]

apply(sim2est,2,sd)
sim2d=sweep(sim2est,2,p0,"-")
colMeans(sim2d)
colMeans(sim2bias)

repl
colMeans(sim2)
mse=colMeans(sim2d^2); mse

pairs(sim2est)


library(GGally)
my_points <- function(data, mapping,...) {
  ggally_points(data = data, mapping = mapping,...) +
    geom_density_2d()
}
my_points <- function(data, mapping) {
  ggplot(data = data, mapping = mapping) +
    geom_point() +
    geom_density_2d()
}
sim2df=as.data.frame(sim2[,1:npar])
columnLabels=c("alpha","beta","gamma",
"sigma","lambda[1]","lambda[2]")

ggpairs(sim2df,columnLabels=columnLabels,
labeller = "label_parsed")
ggpairs(sim2df,lower=list(continuous="density"),
columnLabels=columnLabels,labeller = "label_parsed")
ggpairs(sim2df,lower=list(continuous=my_points),
columnLabels=columnLabels,labeller = "label_parsed")


# Parent Density Single -------------------
```

```
B=100

# choose one set:
alpha=5 ; beta=2 ; gama=7 ; sigma=1 ;
lambda1=5; lambda2=1

alpha=2; beta=2 ; gama=4 ; sigma=0.05 ;
lambda1=2 ; lambda2=2
alpha=1 ; beta=2 ; gama=4 ; sigma=1 ;
lambda1=5; lambda2=1
alpha=1 ; beta=3 ; gama=4 ; sigma=1 ;
lambda1=5 ; lambda2=1
alpha=2; beta=2 ; gama=4 ; sigma=0.9 ;
lambda1=2 ; lambda2=2


p0=c(alpha=alpha,beta=beta,gama=gama,sigma=sigma,
lambda1=lambda1,lambda2=lambda2)
a=alpha ; b=beta ; g=gama ; s=sigma ;
l1=lambda1 ; l2=lambda2
c0=c_func(alpha,beta,gama,sigma,lambda1,lambda2)
c0

# Then:
x=seq(0,1,length=B)
y=seq(0,1,length=B)
f=outer(x,y,dgbkb2,alpha=alpha,beta=beta,
gama=gama,sigma=sigma,lambda1=lambda1,
lambda2=lambda2,c0=c0)
par(mfrow=c(1,1))
persp(x,y,f,
      theta=30,phi=30,expand=0.5,
      col="lightgreen",ltheta=120,
      shade=0.75,ticktype="detailed"
)
contour(x,y,f,xlab="x",ylab="y")
par(mfrow=c(1,1))

p0
integral2(fun=dgbkb2,xmin=0,xmax=1,ymin=0,ymax=1,
alpha=alpha,beta=beta,gama=gama,sigma=sigma,
lambda1=lambda1,lambda2=lambda2,c0=c0)
cubature::hcubature(f=dgbkb,lowerLimit=rep(0,2),
upperLimit=rep(1,2),alpha=alpha,beta=beta,
gama=gama,sigma=sigma,lambda1=lambda1,
lambda2=lambda2,c0=c0,tol=1e-7)

# Marginal -----------------------------

par(mfrow=c(1,2))
curve(
  dgkbX(x, alpha, beta, gama, sigma,
```

```
  lambda1, lambda2, c0),
  from = 0,to = 0.99,  ylab="f(x)",ylim=c(0,5))
curve(
  dgkbX(x, alpha, beta, gama, sigma,
  lambda1, lambda2, c0),
  from = 0,to = 1,  add=TRUE, col="blue")

curve(
  dgkbX(x, alpha, beta, gama, sigma,
  lambda1, lambda2, c0),
  from = 0,to = 0.99,  add=TRUE, col="red")
curve(
  dgkbX(x, alpha, beta, gama, sigma,
  lambda1, lambda2, c0),
  from = 0,to = 0.99,  add=TRUE, col="green")

curve(
  dgkbX(x, alpha, beta, gama, sigma,
  lambda1, lambda2, c0),
  from = 0,to = 1,  add=TRUE, col="orange")

ex<-expression(paste(list(l1==-2,l2==1)),
paste(list(l1==-4,l2==1)),
paste(list(l1==4,l2==1)),
paste(list(l1==1,l2==-3)),
paste(list(l1==7,l2==1)))
legend("top",ex,
col=c("black","red","blue","green","orange"),
cex=0.8,lty=1,text.width = strwidth("1,000,0000"))


ex<-expression(paste(list(alpha==5,beta==2,
gamma==7,sigma==1,l1==5,l2==1)),
paste(list(alpha==2,beta==2,
gamma==4,sigma==0.05,l1==2,l2==2)),
paste(list(alpha==1,beta==2,
gamma==4,sigma==1,l1==5,l2==1)),
paste(list(alpha==1,beta==3, gamma==4,sigma==1,l1==5,l2==1)),
paste(list(alpha==2,beta==2,
gamma==4,sigma==0.9,l1==5,l2==1)))
legend("top",ex,col=c("black","red","blue","green","orange"),
cex=0.8,lty=1,
text.width = strwidth("1,000000000,0000,000000000"))


--------------------------------------------------
curve(
  dgkbY(y, alpha, beta, gama, sigma,
  lambda1, lambda2, c0),
  from = 0,to = 1,
  xname="y",
  ylab="f(y)"
```

```
)

par(mfrow=c(1,1))

integrate(
  f = dgkbX,
  lower = 0,
  upper = 1,
  alpha=alpha,beta=beta,gama=gama,
  sigma=sigma,lambda1=lambda1,lambda2=lambda2,
  c0=c0
)
integrate(
  f = dgkbY,
  lower = 0,
  upper = 1,
  alpha=alpha,beta=beta,gama=gama,
  sigma=sigma,lambda1=lambda1,lambda2=lambda2,
  c0=c0
)


######################################
library(pracma)
dgbkb0<-function(x,y,alpha,beta,gama,sigma,
lambda1,lambda2){
  a=alpha ; b=beta ; g=gama ; s=sigma ;
  l1=lambda1 ; l2=lambda2
  x^(a-1)*y^(b-1)*(1-x)^(g-a-1)*(1-y)^(g-b-1)/
    (1-s*x*y)^g*exp(-(l1*x+l2*y))
}

c_func=function(alpha,beta,gama,sigma,
lambda1,lambda2){
  c0=integral2(fun=dgbkb0,xmin=0,
  xmax=1,ymin=0,ymax=1,
               alpha=alpha,beta=beta,
               gama=gama,sigma=sigma,
               lambda1=lambda1,lambda2=lambda2)$Q
  c0
}
-------------------------------------------
dgbkb<-function(xy,alpha,beta,gama,
sigma,lambda1,lambda2,c0){
  a=alpha ; b=beta ; g=gama ; s=sigma ;
  l1=lambda1 ; l2=lambda2
  x=xy[1]; y=xy[2]
  f<-ifelse(
    all(xy > 0 & xy < 1),
    1/c0*dgbkb0(x,y,alpha,beta,gama,
    sigma,lambda1,lambda2),
    0
```

```
  )
  return(f)
}
-------------------------------------------
dgbkb2<-function(x,y,alpha,beta,gama,sigma,
lambda1,lambda2,c0) {
  a=alpha ; b=beta ; g=gama ; s=sigma ;
  l1=lambda1 ; l2=lambda2
  f=1/c0*dgbkb0(x,y,alpha,beta,gama,
  sigma,lambda1,lambda2)
  return(f)
}
---------------------------------------------------------

# choose one set:
alpha=2 ; beta=2 ; gama=4 ; sigma=0.5 ;
lambda1=1 ; lambda2=1
# alpha=2 ; beta=2 ; gama=6 ; sigma=0.5 ;
# lambda1=2 ; lambda2=2
# alpha=2 ; beta=2 ; gama=4 ; sigma=0.5 ;
# lambda1=1.5 ; lambda2=1.5

p0=c(alpha=alpha,beta=beta,gama=gama,
sigma=sigma,lambda1=lambda1,lambda2=lambda2)
p0
a=alpha ; b=beta ; g=gama ; s=sigma ;
l1=lambda1 ; l2=lambda2
c0=c_func(alpha,beta,gama,sigma,lambda1,lambda2)
c0
--------------------------------------------------
# Then:
B=100
x=seq(0,1,length=B)
y=seq(0,1,length=B)
f=outer(x,y,dgbkb2,alpha=alpha,beta=beta,
gama=gama,sigma=sigma,lambda1=lambda1,
lambda2=lambda2,c0=c0)
#par(mfrow=c(1,2))
persp(x,y,f,
      theta=30,phi=30,expand=0.5,
      col="lightgreen",ltheta=120,
      shade=0.75,ticktype="detailed"
)
contour(x,y,f,xlab="x",ylab="y")
par(mfrow=c(1,1))

p0
integral2(fun=dgbkb2,xmin=0,xmax=1,ymin=0,
ymax=1,alpha=alpha,beta=beta,gama=gama,
sigma=sigma,lambda1=lambda1,lambda2=lambda2,c0=c0)
-----------------------------------------------
```

```
#marginal plot
---------------------------------------------
p00=function(x){
h=function(y) y^(beta-1)*(1-y)^(gama-beta-1)
*exp(-lambda2*y)*(1-sigma*x*y)^(-gama)

integrate(dgbkb2,0,1,alpha=alpha,beta=beta,
gama=gama,sigma=sigma,lambda1=lambda1,
lambda2=lambda2,c0=c0 )$value
}
p1=Vectorize(p00,"x")
x=seq(0,1,by=0.01)
cbind(x,p1=p1(x))


h=function(y) y^(b-1)*(1-y)^(g-b-1)*exp(-l2*y)*(1-s*x*y)^(-g)
integrate(h,0,1)


B=100
alpha=2 ; beta=2 ; gama=4 ; sigma=0.5 ;
lambda1=1 ; lambda2=1
a=alpha ; b=beta ; g=gama ; s=sigma ;
l1=lambda1 ; l2=lambda2
dgbkb0<-function(x,y,alpha,beta,gama,
sigma,lambda1,lambda2){
  a=alpha ; b=beta ; g=gama ;
  s=sigma ; l1=lambda1 ; l2=lambda2
  x^(a-1)*y^(b-1)*(1-x)^(g-a-1)*(1-y)^(g-b-1)/
    (1-s*x*y)^g*exp(-(l1*x+l2*y))
}

c_func=function(alpha,beta,gama,sigma,
lambda1,lambda2){
  c0=integral2(fun=dgbkb0,xmin=0,
  xmax=1,ymin=0,ymax=1,
  alpha=alpha,beta=beta,gama=gama,
  sigma=sigma,lambda1=lambda1,lambda2=lambda2)$Q
  c0
}
c0

alpha=2 ; beta=2 ; gama=4 ; sigma=0.5 ;
lambda1=1 ; lambda2=1
a=alpha ; b=beta ; g=gama ; s=sigma ;
l1=lambda1 ; l2=lambda2

x=seq(0,1,0.01)
x
a=rep(0,101)
a
h=function(y) y^(b-1)*(1-y)^(g-b-1)*exp(-l2*y)*(1-s*x*y)^(-g)
```

```
for(i in 1:101)
a[i]=integrate(h,0,1)

a
curve(f,0,1)

curve(f,0,1)
```
